# *Staphylococcus Aureus* Tames Nociceptive Neurons to Suppress Synovial Macrophage Responses for Sustained Infection in Septic Arthritis

**DOI:** 10.1002/advs.202409251

**Published:** 2025-02-17

**Authors:** Xinyu Fang, Yang Chen, Haiqi Ding, Changyu Huang, Hongxin Hu, Chaofan Zhang, Yunzhi Lin, Qijin Wang, Xueni Hu, Yiming Lin, Yongfa Chen, Nanxin Zhang, Xuhui Yuan, Ying Huang, Wenbo Li, Susheng Niu, Jianhua Lin, Bin Yang, Tifei Yuan, Wenming Zhang

**Affiliations:** ^1^ Department of Orthopedic Surgery, National Regional Medical Center, Binhai Campus of the First Affiliated Hospital Fujian Medical University Fuzhou 350000 China; ^2^ Department of Orthopedic Surgery The First Affiliated Hospital of Fujian Medical University Fuzhou 350000 China; ^3^ Fujian Provincial Institute of Orthopedics the First Affiliated Hospital, Fujian Medical University Fuzhou 350000 China; ^4^ Department of Stomatology, The First Affiliated Hospital Fujian Medical University Fuzhou 350000 China; ^5^ Department of Laboratory Medicine The First Affiliated Hospital of Fujian Medical University Fuzhou 350000 China; ^6^ Key Laboratory of Orthopedics & Traumatology of Traditional Chinese Medicine and Rehabilitation Ministry of Education Fujian University of Traditional Chinese Medicine Fuzhou 350000 China; ^7^ Shanghai Key Laboratory of Psychotic Disorders, Brain Health Institute, National Center for Mental Disorders, Shanghai Mental Health Center Shanghai Jiaotong University School of Medicine and School of Psychology Shanghai 200000 China; ^8^ Co‐innovation Center of Neuroregeneration Nantong University Nantong Jiangsu 226019 China

**Keywords:** calcitonin gene‐related peptide, chronic septic arthritis, macrophages, nociceptive neuron, *Staphylococcus aureus*

## Abstract

The interaction between the nervous system and immune system during chronic bacterial infection remains unclear. Here, it is reported that *Staphylococcus aureus* (*S. aureus*) infection induces calcitonin gene‐related peptide (CGRP) secretion from intra‐articular transient receptor potential cation channel subfamily V member 1 positive (TRPV1^+^) nociceptive nerves through its pore‐forming toxin (PFT) α‐hemolysin. The released CGRP then inhibits the production of chemotactic cytokines by CX3CR1^+^ tissue‐resident synovial lining macrophages via receptor activity modifying protein 1 (RAMP1) receptors at the onset of septic arthritis. During the subsequent chronic course of infection, the continuous release of CGRP triggered by pain has a lasting effect on the antimicrobial capabilities of macrophages, thereby promoting bacterial survival and joint damage. This evidence suggests a critical role for neuroimmune regulation in *S. aureus‐*induced chronic septic arthritis. CGRP receptor antagonism may reduce joint destruction, thus providing a new option for treating bone and joint infections.

## Graphical abstract

*Staphylococcus aureus* (*S. aureus*) infection induces persistent pain via toxins, leading to CGRP release from TRPV1^+^ nociceptive nerves. CGRP inhibits chemotactic factor release from CX3CR1^+^ synovial lining macrophages and suppresses their bactericidal activity. This study highlights neuroimmune interactions in chronic septic arthritis and suggests CGRP receptor antagonism as a novel therapeutic strategy.

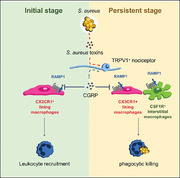

## 1 Introduction

The gram‐positive bacterium *Staphylococcus aureus* (*S. aureus*) is a leading cause of skin and soft tissue infections in humans. The prevalence of its methicillin‐resistant strain (MRSA) has been steadily increasing, making it a serious public health problem. Given that it is difficult to achieve effective antibiotic concentrations in deep infections of closed joints, septic arthritis is the most difficult *S. aureus* infection to cure,^[^ ^1^, ^2^ ^]^ with an incidence of ≈4–29 cases per 100 000 people and a mortality rate of more than 10%.^[^ ^1^, ^2^, ^3^, ^4^ ^]^ Even after thorough surgical treatments combined with antibiotics, many patients are prone to relapse and develop chronic infections.^[^ ^5^ ^]^ Although new effective antibiotics are constantly being developed, the renewal rate of antibiotics still fails to meet clinical needs. Therefore, there is an urgent need to enhance our understanding of the mechanisms of *S. aureus* resistance to the existing treatment methods for septic arthritis to facilitate the development of effective treatments.

Infection with *S. aureus* can cause severe pain at the infected site.^[^ ^6^ ^]^ Nociceptive neurons are specific subsets of peripheral sensory neurons that mediate pain, a sensation that warns the body of potential danger.^[^ ^7^ ^]^ In addition to transmitting pain signals to the central nervous system, nociceptors release neuropeptides from peripheral nerve endings, which directly regulate inflammation. These neuropeptides bind to their homologous receptors on immune cells, resulting in changes in transcription, cytokine production, and immune cell phenotypes.^[^ ^8^ ^]^ Joint nociceptive neurons also actively communicate with the immune system. For example, in inflammatory arthritis, capsaicin‐sensitive transient receptor potential cation channel subfamily V member 1 (TRPV1)‐positive neurons regulate the distribution of immune cells and the intensity of inflammation.^[^ ^9^ ^]^

Recent studies have indicated that in cases of acute microbial infections of immune barriers, such as the skin and lungs, sensory neurons interact with immune cells and can suppress bactericidal activity through pathways involving bacterial toxins, neuropeptides, and neutrophils.^[^ ^10^, ^11^, ^12^ ^]^ Cutaneous *S. aureus* infection may rapidly elicit a robust immune response, including the formation and rupture of abscesses, which can eventually resolve. However, in the absence of treatment, *S. aureus*‐induced septic arthritis typically results in a persistent infectious state.^[^ ^5^, ^13^, ^14^ ^]^ In this more persistent immune response mode of nonbarrier organs, prolonged interaction between *S. aureus* and nociceptors may influence the host's immune response differently during chronic septic arthritis.

Thus, we collected clinical *S. aureus* strains from septic arthritis patients and found that *hla* (encoding α‐hemolysin) gene expression was elevated in patients with severe pain. We found that the *S. aureus* pore‐forming toxin (PFT) α‐hemolysin (Hla) directly stimulated TRPV1^+^ nociceptors in the knee synovium at the onset of infection, during which calcitonin gene‐related peptide (CGRP) was released toward the RAMP1 receptors of CX3CR1^+^ tissue‐resident synovial lining macrophages and inhibited chemotactic cytokine release. During the persistent stage of infection, the continuous release of CGRP initiated by pain exerted a lasting impact on the bactericidal ability of macrophages through RAMP1 receptors and established a niche favorable for bacterial survival. This evidence confirms the sustained action of the nervous system in host immune regulation during *S. aureus*‐induced septic arthritis and the possibility of regulating the immune response to chronic infection by blocking neuropeptides.

## 2 Results

### 2.1 Nociceptors Regulate Septic Arthritis

**Figure 1:**
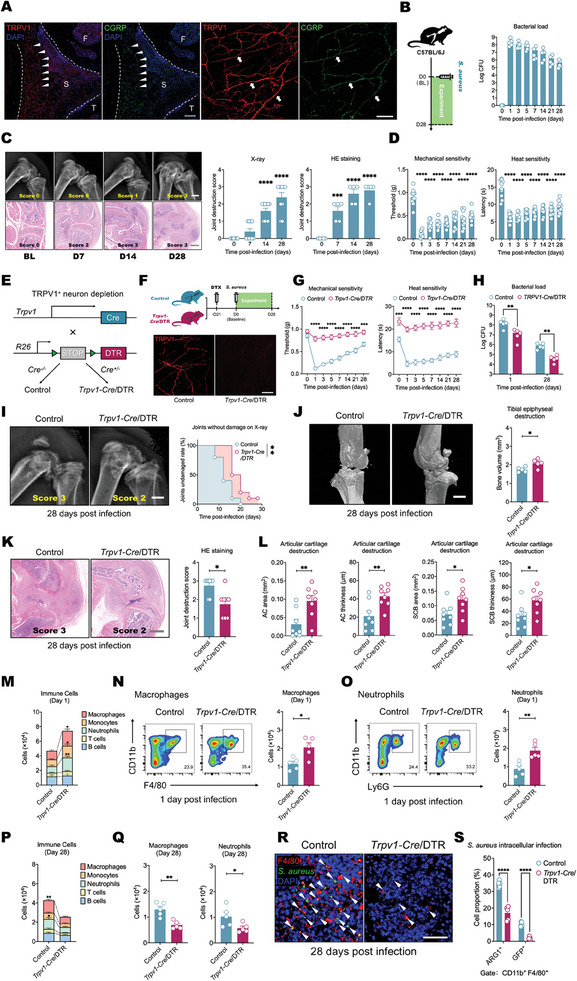
TRPV1^+^ nociceptors that mediate pain inhibit the defense against *S. aureus* in the host's joint. A) Sagittal section (left) and whole‐mount (right) confocal images of the knee joint synovium showing abundant innervating TRPV1^+^ and CGRP^+^ nociceptive nerves (arrows) on the synovium (arrowheads and arrows: nociceptive nerves; F: femur; T: tibia; S: synovium; scale bar: 100 µm). B) Temporal changes in the bacterial load recovered from the knee joints of WT mice infected with *S. aureus* (1 × 10^8^ colony‐forming units (CFUs)). The time point labeled ‘0′ signifies the baseline condition prior to the onset of infection. *n* = 5 mice/group. C) X‐ray (above) and H&E staining images (below) showing the progression of knee joint damage in WT mice infected with *S. aureus*. The time point labeled ‘0′ signifies the baseline condition prior to the onset of infection. *n* = 5 mice/group. (Scale bar: 500 µm). D) Temporal changes in mechanical (left) and thermal (right) hyperalgesia in WT mice infected with *S. aureus*. The time point labeled ‘0′ signifies the baseline condition prior to the onset of infection. *n* = 10 mice/group. E) Generation of *Trpv1‐Cre/*DTR and littermate control mice. F) Intra‐articular injection of DTX (10 ng/5 µl) into *Trpv1‐Cre/*DTR or control mice (above). Synovium innervation by TRPV1^+^ neurons after local application of DTX in control and *Trpv1‐Cre/*DTR mice (below). (Scale bar: 100 µm). G) Differences in mechanical (left) and thermal (right) hyperalgesia after *S. aureus* (1 × 10^8^ CFU) infection between control and *Trpv1‐Cre/*DTR mice treated locally with DTX. The time point labeled ‘0′ signifies the baseline condition prior to the onset of infection. *n* = 10 mice/group. H) Bacterial load in samples collected on the 1st day and 28th day postinjection of *S. aureus* from both the control group and the *Trpv1‐Cre*/DTR mouse group, each treated locally with DTX. *n* = 5 mice/group. I) Typical X‐ray images of knee joints on the 28th day after *S. aureus* infection in control and *Trpv1‐Cre/*DTR mice (left) and survival curves reflecting the differences in joint destruction progression after *S. aureus* infection between the two groups (right). The time point labeled ‘0′ signifies the baseline condition prior to the onset of infection. *n* = 10 mice/group. (Scale bar: 500 µm). J) Typical micro‐CT images of knee joints on the 28th day after *S. aureus* infection in control and *Trpv1‐Cre/*DTR mice (left) and the differences in tibial epiphyseal volume between the two groups (right). *n* = 5 mice/group. (Scale bar: 500 µm). K) Typical H&E‐stained images of knee joints (left) and scores based on H&E staining showing differences in synovial and bone damage between the two groups on the 28th day after *S. aureus* infection (right). *n* = 8 mice/group. (Scale bar: 500 µm). L) Differences in the area and thickness of the articular cartilage (AC area and AC thickness) and subchondral bone (SCB area and SCB thickness) of the tibial plateaus between the two groups on the 28th day after S. aureus infection. *n* = 8 mice/group. M) Flow cytometry quantification of immune cells in the knee joint synovium of control and *Trpv1‐Cre/*DTR mice on the 1st day after *S. aureus* infection. *n* = 5 mice/group. N) Typical flow cytometry images and quantification of synovial macrophages in the knee joints of control and *Trpv1‐Cre/*DTR mice on the 1st day after *S. aureus* infection. *n* = 5 mice/group. O) Typical flow cytometry images and quantification of synovial neutrophils in the knee joints of control and *Trpv1‐Cre/*DTR mice on the 1st day after *S. aureus* infection. *n* = 5 mice/group. P) Flow cytometry quantification of immune cells in the knee joint synovium of control and *Trpv1‐Cre/*DTR mice on the 28th day after *S. aureus* infection. *n* = 5 mice/group. Q) Flow cytometry quantification of synovial macrophages and neutrophils in the knee joints of control and *Trpv1‐Cre/*DTR mice on the 28th day after *S. aureus* infection. *n* = 5 mice/group. R) Typical whole‐mount immunofluorescence staining of macrophages and GFP^+^ *S. aureus* in the knee joint synovium of control and *Trpv1‐Cre/*DTR mice on the 28th day after GFP^+^ *S. aureus* (1 × 10^8^ CFU) infection. (arrowheads: *S. aureus*, Scale bar: 100 µm). S) Flow cytometry quantification of anti‐inflammatory macrophages (ARG1^+^) and intracellular *S. aureus*‐harboring macrophages (GFP^+^) in the knee joint synovium of control and *Trpv1‐Cre/*DTR mice on the 28th day after GFP^+^ *S. aureus* infection. *n* = 5 mice/group. Statistical analysis: G,M,P,S) Two‐way ANOVA, Sidak post hoc test. H,J,L,N,O,Q) Student's t tests. K) Mann‐Whitney U test. I) Restricted mean survival time (RMST). *p < 0.05, **p < 0.01, ***p < 0.001, ****p < 0.0001. ns = not significant. Mean ± SEM.

Septic arthritis is frequently accompanied by intense pain, which is thought to arise from nociceptive nerve endings in the synovium. These nerve endings may directly detect pathogens in the affected joint, playing a central role in generating the pain response. TRPV1 is a voltage‐gated channel expressed by nociceptors that mediates inflammatory pain. CGRP is stored in dense core vesicles at the nerve endings of nociceptors. Through confocal microscopy imaging of the mouse synovium, we confirmed the presence of a dense network of TRPV1^+^ and CGRP^+^ nerves in the synovium (**Figure** **1**A; Figure S1A, Supporting Information). Human septic arthritis typically arises from the introduction of colonizing bacteria, most commonly *S. aureus*, into the joint through invasive procedures.^[^ ^15^ ^]^ This condition often follows a chronic course and is difficult to cure. To simulate this infection, we injected *S. aureus* into the mouse joint cavity (Figure 1B). The synovium is generally considered a defensive barrier within the joint.^[^ ^16^ ^]^ By isolating the synovium, we analyzed the temporal progression of *S. aureus* invasion into the joint. *S. aureus* could be isolated from the joint synovium throughout the 0‐ to 28‐day period post infection (Figure 1B). Continuous tracking via X‐ray and hematoxylin and eosin (H&E) staining of tissue sections revealed that the natural proliferation of *S. aureus* within the synovium of most wild‐type (WT) mice led to progressive synovial damage, which typically progressed to bone erosions and defects 14 days after infection (Figure 1C). The persistent state of infection evoked prolonged pain hypersensitivity (Figure 1D). The presence of TRPV1^+^ and CGRP^+^ nociceptive nerves in synovial tissue during chronic infection was revealed by immunofluorescence staining, providing the structural basis for persistent pain (Figure S1B,C, Supporting Information).

To determine the role of TRPV1^+^ nociceptors in host defense, we bred *Trpv1‐Cre* mice with DTR^flox^ mice, in which Cre induced the expression of diphtheria toxin receptor (DTR) in TRPV1^+^ neurons (Figure 1E). Intra‐articular injection of diphtheria toxin (DTX) selectively ablated TRPV1^+^ nociceptors in the synovium of *Trpv1‐Cre/*DTR mice. Immunofluorescence confirmed the elimination of nociceptors in the synovium of the *Trpv1‐Cre/*DTR mice following DTX injection compared with the Cre^−^ littermate controls (Figure 1F). When infected with *S. aureus*, *Trpv1‐Cre/*DTR mice exhibited less pain hypersensitivity at various time points than did control mice (Figure 1G). Concurrently, on days 1 and 28 postinfection, the *Trpv1‐Cre/*DTR mice had lower bacterial loads isolated from the synovium than did the control mice (Figure 1H). Continuous X‐ray imaging was used to track the progression of septic arthritis, with a focus on reaching the endpoint of visible bone damage for survival analysis (Figure 1I). Compared with the control group, the *Trpv1‐Cre/*DTR group exhibited markedly longer mean bone survival (free of damage) (Figure 1I). After 28 days of infection, further evaluation of synovial and bone damage was performed through HE staining and micro‐CT. The degree of joint damage in *Trpv1‐Cre/*DTR mice was markedly reduced (Figure 1J–L). As an alternative approach to ablating nociceptors, we injected resiniferatoxin (RTX), a specific TRPV1 agonist, into mouse joints to induce local neuronal loss (Figure S1D, Supporting Information). Compared with control mice, RTX‐treated mice exhibited attenuated pain hypersensitivity and joint bacterial loads following *S. aureus* infection (Figure S1E,F, Supporting Information). Compared with the control treatment, RTX treatment also delayed bone erosion and ameliorated joint damage at 28 days post infection (Figure S1G–J, Supporting Information).

We hypothesized that TRPV1^+^ nociceptors may influence immune responses during septic arthritis. *Trpv1‐Cre/*DTR and control mice showed comparable baseline numbers of synovial leukocytes (Figure S1K, Supporting Information). At 24 h postinfection with *S. aureus*, the *Trpv1‐Cre/*DTR mice exhibited a more pronounced increase in intra‐articular leukocyte numbers than did the control mice (Figure 1M). Specifically, the number of myeloid cells (macrophages, neutrophils, and monocytes) significantly differed, whereas the number of lymphocytes (B cells and T cells) did not significantly differ (Figure 1M–O; Figure S1L, Supporting Information). Additionally, there were no differences in number of lymphocytes between the two groups on day 7 post‐infection (Figure S1M, Supporting Information). Notably, macrophages and neutrophils, crucial innate immune responders after infection, were significantly more abundant in the synovium in the *Trpv1‐Cre/*DTR mice than in the control mice (Figure 1N,O).

During the chronic phase (28 days postinfection), there was a reversal in immune cell recruitment, with markedly diminished immune cell infiltration observed in *Trpv1‐Cre/*DTR mice, whereas relatively greater numbers of immune cells, chiefly macrophages and neutrophils, remained present in the joints of control mice (Figure 1P,Q). The persistent recruitment of neutrophils and macrophages in control mice may have been due to poor containment of local infection, which induced continuous activation of the immune system. However, the sustained recruitment of immune cells did not reduce the bacterial load (Figure 1H). Whole‐mount immunofluorescence staining of the synovium revealed that, in the control group, a greater number of macrophages exhibited colocalization of GFP‐labeled *S. aureus* within their cytoplasm (Figure 1R). Electron microscopy further confirmed the intracellular retention of *S. aureus* within macrophages in the control group (Figure S1N, Supporting Information), whereas this phenomenon was significantly reduced in *Trpv1‐Cre/*DTR mice (Figure 1R; Figure S1N, Supporting Information). Further flow cytometry sorting of the joint synovium revealed more arginase 1 (ARG1)‐positive anti‐inflammatory macrophages in control mice than in *Trpv1‐Cre/*DTR mice (Figure 1S). Compared with ARG1‐ macrophages, a greater percentage of ARG1^+^ macrophages were GFP^+^ (Figure S1O, Supporting Information), indicating their role in promoting the intracellular survival of *S. aureus*. This resulted in a markedly increased percentage of GFP^+^ macrophages in the control mice (Figure 1S). During the process of sustained infection, TRPV1^+^ nociceptors may shift macrophages toward an anti‐inflammatory phenotype with compromised bactericidal capacity, providing favorable conditions for intracellular bacterial survival.

### 2.2 Bacteria Directly Trigger Long‐Term Release of CGRP in the Synovium

**Figure 2:**
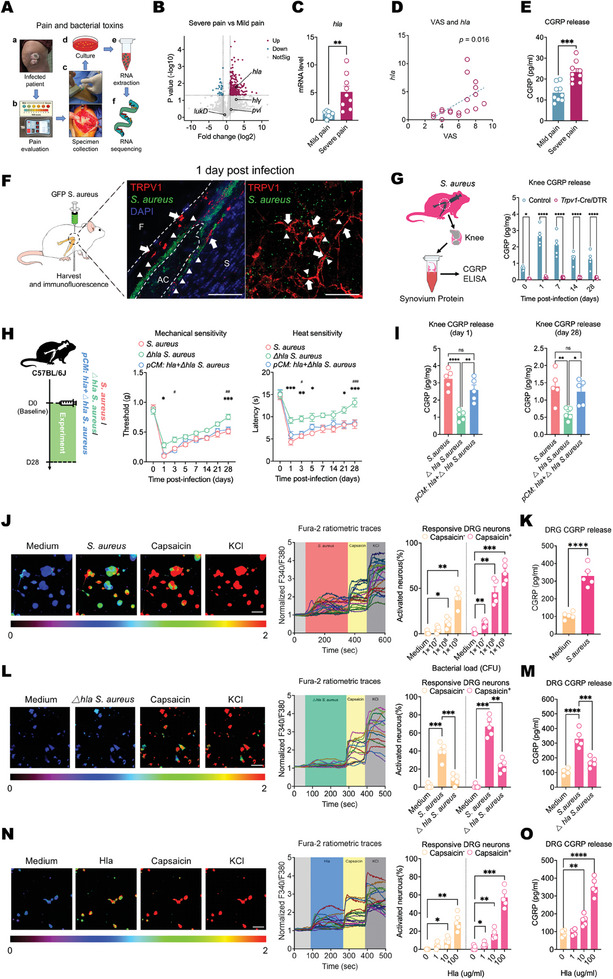
*S. aureus* induces synovial nociceptor activation and sustains CGRP release through Hla. A) Schematic diagram showing the clinical evaluation process, isolation of bacteria by culture, and sequencing of the isolated *S. aureus* strains from patients with septic arthritis. B) Volcano plot showing differentially expressed genes in *S. aureus* strains isolated from patients with severe pain (VAS score>6) compared with those isolated from patients with mild pain (VAS score≤6). A gene is considered significantly upregulated or downregulated if its expression is greater than 2‐fold or less than 0.5‐fold in the severe pain group compared to the mild pain group, along with a P value < 0.05. Key thresholds are indicated by dashed lines. Upregulated, downregulated, and nonsignificantly changed genes are represented by red, blue, and gray dots, respectively. *n* = 9 strains/group. C) qRT‐PCR analysis confirmed significant differences in *hla* gene expression between *S. aureus* strains isolated from the two groups of patients. *n* = 9 strains/group. D) Assessment of the correlation between patient VAS scores and *hla* gene expression in isolated *S. aureus* strains. *n* = 18, p = 0.016. E) Differences in CGRP expression in synovial fluid between the two groups of patients. *n* = 9 patients/group. F) Establishment of the septic arthritis model by injecting GFP^+^ *S. aureus* into the knee joints of mice (left). Immunofluorescence staining of knee joint sagittal sections (middle) and synovial whole‐mount (right) shows the spatial relationship between *S. aureus* and nociceptive nerves (arrows: nociceptive nerves; arrowheads: *S. aureus*; F: femur; AC: articular cavity; S: synovium). (Scale bar: 100 µm). G) ELISA examination of CGRP expression in the knee joint synovium of the control group and *Trpv1‐Cre/*DTR mice on days 0 (uninfected), 1, 7, 14, and 28 after *S. aureus* (1 × 10^8^ CFU) infection. *n* = 5 mice/group. H) Differences in mechanical (left) and thermal (right) hyperalgesia among *S. aureus*‐infected mice, *Δhla*‐*S. aureus*‐infected mice and *pCM: hla*+*△hla*‐*S. aureus*‐infected mice. The time point labeled “0′ signifies the baseline condition prior to the onset of infection. *n* = 10 mice/group^−1^. I) Differences in CGRP expression in the synovium among *S. aureus*‐infected mice, *Δhla*‐*S. aureus*‐infected mice and *pCM: hla*+*△hla*‐*S. aureus*‐infected mice on the 1st day (left) and 28th day (right) after infection. *n* = 5 mice group^−1^. J) Calcium imaging of DRG neurons stimulated with *S. aureus* (1 × 10^9^ CFU), capsaicin (1 µM) and KCl (40 mM) showing typical fields of view (left) and calcium traces (middle). (Scale bar: 50 µm; color bar, F340/F380 ratio). The right graph represents the proportions of capsaicin‐nonresponsive and capsaicin‐responsive neurons activated by different amounts of *S. aureus* (1 × 10^7^, 1 × 10^8^, or 1 × 10^9^ CFU). *n* = 5 wells group^−1^. K) ELISA examination of CGRP release in the cell culture supernatants of DRG neurons stimulated with medium and *S. aureus* for 30 min. *n* = 5 wells group^−1^. L) Calcium imaging of DRG neurons stimulated with *Δhla*‐*S. aureus* (1 × 10^9^ CFU), capsaicin (1 µM) and KCl (40 mM) showing typical fields of view (left) and calcium traces (middle). (Scale bar: 50 µm; color bar, F340/F380 ratio). The right graph represents the proportions of capsaicin‐nonresponsive and capsaicin‐responsive neurons activated by *Δhla‐S. aureus* (1 × 10^9^ CFU) and WT *S. aureus* (1 × 10^9^ CFU). *n* = 5 wells group^−1^. M) ELISA examination of CGRP release in the cell culture supernatants of DRG neurons stimulated with *Δhla‐S. aureus* and WT *S. aureus* for 30 min. *n* = 5 wells group^−1^. N) Calcium imaging of DRG neurons stimulated with Hla (100 µg ml^−1^), capsaicin (1 µM) and KCl (40 mM) showing typical fields of view (left) and calcium traces (middle). (Scale bar: 50 µm; color bar, F340/F380 ratio). The right graph shows the proportions of capsaicin‐nonresponsive and capsaicin‐responsive neurons activated by different concentrations of Hla (1, 10, or 100 µg ml^−1^). *n* = 5 wells group^−1^. O) ELISA of CGRP release in the cell culture supernatants of DRG neurons stimulated with different concentrations of Hla (1, 10, or 100 µg ml^−1^) for 30 min. *n* = 5 wells group^−1^. Statistical analysis: C,E,K) Student”s t tests. D) Spearman rank test. G,H) Two‐way ANOVA, Sidak post hoc test. I,J,L,M,N,O) One‐way ANOVA, Dunnett post hoc test. *p < 0.05, **p < 0.01, ***p < 0.001, ****p < 0.0001. ns = not significant. Mean ± SEM. In Figure H, the *S. aureus* group versus the *Δhla S. aureus* group, *p < 0.05, **p < 0.01, ***p < 0.001, ****p < 0.0001, the *Δhla S. aureus* group versus the *pCM: hla* +*△hla S. aureus* group, ^#^ *p* < 0.05, ^##^ *p* < 0.01, ^###^ *p* < 0.001, ^####^ *p* < 0.0001.

To investigate whether pain in clinical patients is associated with bacterial features, we enrolled patients with *S. aureus*‐induced septic arthritis and conducted pain assessments along with bacterial prokaryotic transcriptome sequencing (**Figure** **2**A). The results revealed a significantly greater expression level of the *hla* gene, encoding α‐hemolysin, in *S. aureus* isolates obtained from severe pain patients (visual analog scale [VAS>6]) than in those from mild pain patients (VAS≤6) (Figure 2B). We validated the objectivity of the VAS scores via pressure‒pain threshold (PPT) testing (Figure S2A–C, Supporting Information). The grouping based on VAS scoring was performed according to previous studies.^[^ ^17^, ^18^, ^19^ ^]^ This finding was further confirmed by qRT‐PCR analysis (Figure 2C; Figure S2D, Supporting Information). Additionally, a linear correlation was observed between the expression levels of the *hla* gene and pain scores (Figure 2D). These findings indicate that the expression level of the *hla* gene can serve as an indicator of pain severity in patients. Among the inflammation markers assessed, only C‐reactive protein (CRP) was different between the groups, and none of the inflammation markers showed a linear correlation with the VAS scores (Figure S2E–L, Supporting Information). Further analysis revealed that patients with higher pain grades presented corresponding increases in the expression levels of CGRP in the synovial fluid (Figure 2E).

We isolated synovium from uninfected mice and sorted CD45^+^ synovial cells for single‐cell RNA sequencing (scRNA‐seq) analysis to determine which neuropeptides synovial immune cells may respond to. The single‐cell UMAP plot revealed distinct clusters of immune cells in the synovium, with macrophages and neutrophils being the most abundant populations (Figure S2M, Supporting Information). Receptor activity modifying protein 1 *Ramp1* and calcitonin‐receptor‐like receptor *Calcrl* were the top‐ranked neuropeptide receptor genes in the list of genes expressed in synovial immune cell clusters (Figure S2N, Supporting Information). Other expressed neuropeptide receptor genes included *Ramp2*, *Ramp3*, *Vipr1*, *Tacr1*, *Avpr2*, *Npr1*, and *Bdkrb2* (Figure S2N, Supporting Information). Because *Ramp1* and *Calcrl* genes were closely associated with the expression of CGRP receptors, we focused on neuropeptide CGRP in subsequent parts of this study.

In contrast to prior studies that solely examined changes in CGRP release during acute infection,^[^ ^20^ ^]^ our investigation included mice infected for nearly one month. During both the acute and chronic phases of infection, we identified the colocalization of GFP‐labeled *S. aureus* with TRPV1^+^ nerves in the synovium, indicating persistent contact between the bacteria and the nerves (Figure 2F; Figure S3A, Supporting Information). The levels of CGRP in the synovium were significantly elevated postinfection compared with those in uninfected mice (day 0), whereas *Trpv1‐Cre/*DTR mice exhibited a notable reduction in CGRP release compared with WT‐infected mice, suggesting that septic arthritis triggers CGRP release, with TRPV1^+^ nociceptors being the primary source (Figure 2G). We then examined the role of Hla in pain and CGRP release by establishing an animal model of infection with *hla* gene‐knockout *S. aureus* (*Δhla S. aureus*). Hyperalgesia persisted in the *S. aureus*‐infected mice throughout the 28‐day infection period but was alleviated in the *Δhla S. aureus*‐infected mice (Figure 2H). Hyperalgesia was not further reduced when *Δhla S. aureus* was used to infect *Trpv1‐Cre*/DTR mice (Figure S3B, Supporting Information). *Δhla S. aureus*‐infected mice also presented decreased synovial CGRP release during both the initial (1 day) and persistent (28 day) phases of infection (Figure 2I). Plasmid complementation with a functional copy of *hla* (*pCM*: *hla*) fully restored the hyperalgesia and CGRP release induced by the *Δhla S. aureus* strains (Figure 2H,I). The pain hypersensitivity induced in WT mice by recombinant Hla was significantly attenuated in *Trpv1‐Cre*/DTR mice (Figure S3C, Supporting Information). We found that the deficiency of the *hla* gene does not affect the growth curve of *S. aureus* (Figure S3D, Supporting Information). Although there was a reduction in bacterial load and joint destruction following *S. aureus* infection in the *hla* gene deficiency group, the differences were not statistically significant (Figure S3E–G, Supporting Information). We propose that this may be due to the fact that the reduction in Hla stimulation does not eliminate the influence of nociceptive nerves on infection completely, as the deficiency in nociceptive nerves does.

Next, we explored whether the release of CGRP in the synovium following bacterial infection is directly associated with the activation of nociceptive neurons. Fluorogold (FG) retrograde tracing revealed the presence of neurons innervating the knee in the L3‐5 dorsal root ganglia (DRGs) (Figure S3H,I, Supporting Information). In vitro cultures of these DRGs exhibited calcium influx responses upon stimulation with *S. aureus*, with higher bacterial concentrations activating a greater number of neurons (Figure 2J). Most neurons responding to bacterial stimulation also responded to the TRPV1 ligand capsaicin (Figure 2J). Furthermore, *S. aureus* stimulation induced CGRP release from DRG neurons (Figure 2K). Calcium influx and CGRP release were significantly lower in response to *Δhla S. aureus* than in response to WT *S. aureus* (Figure 2L,M). Recombinant Hla dose‐dependently induced calcium influx and CGRP release in DRG neurons (Figure 2N,O). Plasmid complementation with a functional copy of *hla* (*pCM*: *hla*) in the strains restored the ability of *S. aureus* to activate neurons at levels similar to those of WT bacteria (Figure S3J,K, Supporting Information). These data suggest that *S. aureus* can directly and persistently activate nociceptors and that CGRP is released in the synovium through toxin‐mediated mechanisms.

### 2.3 CGRP Suppresses Synovial Myeloid Cells to Influence Infection

**Figure 3:**
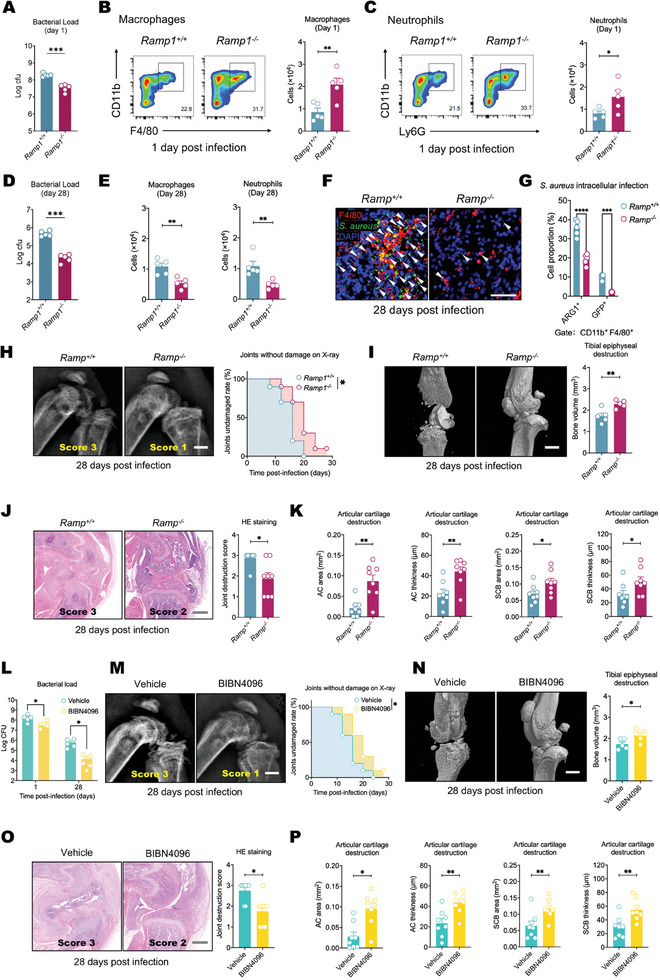
Blocking CGRP‐Ramp1 signaling in myeloid cells enhances synovial responses to *S. aureus* infection. A) Differences in the local bacterial load in the knee between control and *Ramp1* ^−/−^ mice on the 1st day post infection (*S. aureus*, 1 × 10^8^ CFU). *n* = 5 mice group^−1^. B) Representative flow cytometry images and quantification of macrophages in the knee synovium of control and *Ramp1* ^−/−^ mice on the 1st day post infection. *n* = 5 mice group^−1^. C) Representative flow cytometry images and quantification of neutrophils in the knee synovium of control and *Ramp1^−/−^* mice on the 1st day post infection. *n* = 5 mice group^−1^. D) Local bacterial load differences between control and *Ramp1* ^−/−^ mice on the 28th day post infection. *n* = 5 mice/group. E) Quantification of macrophages (left) and neutrophils (right) in the knee synovium of control and *Ramp1* ^−/−^ mice on the 28th day post infection. *n* = 5 mice group^−1^. F) Typical whole‐mount immunofluorescence staining of macrophages and GFP^+^ *S. aureus* in the knee joint synovium of control and *Ramp1* ^−/−^ mice on the 28th day after infection (GFP^+^ *S. aureus*, 1 × 10^8^ CFU). (arrowheads: *S. aureus*, Scale bar: 50 µm). G) Flow cytometry quantification of anti‐inflammatory macrophages (ARG1^+^) and intracellular *S. aureus*‐harboring macrophages (GFP^+^) in the knee joint synovium of control and *Ramp1* ^−/−^ mice on the 28th day after infection. *n* = 5 mice/group^−1^. H) Typical X‐ray images of knee joints on the 28th day after *S. aureus* infection in control and *Ramp1* ^−/−^ mice (left) and survival curves reflecting the differences in bone destruction progression between the two groups (right). The time point labeled ‘0′ signifies the baseline condition prior to the onset of infection. *n* = 10 mice/group^−1^. (Scale bar: 500 µm). I) Typical micro‐CT images of knee joints on the 28th day after *S. aureus* infection in control and *Ramp1* ^−/−^ mice (left) and the differences in tibial epiphyseal volume between the two groups (right). *n* = 5 mice group^−1^. (Scale bar: 500 µm). J) Typical H&E‐stained images of knee joints (left) and scores based on H&E staining showing differences in synovial and bone damage between control and *Ramp1* ^−/−^ mice on the 28th day after *S. aureus* infection (right). *n* = 8 mice group^−1^. (Scale bar: 500 µm). K) Differences in the area and thickness of the articular cartilage (AC area and AC thickness) and subchondral bone (SCB area and SCB thickness) of the tibial plateaus between the two groups on the 28th day after S. aureus infection. *n* = 8 mice group^−1^. L) Bacterial load in the knee joints of mice treated with vehicle or BIBN4096 on the 1st day and 28th day post infection. *n* = 5 mice group^−1^. M) Typical X‐ray images of knee joints on the 28th day after *S. aureus* infection in vehicle and BIBN4096 treated mice (left) and survival curves reflecting the differences in bone destruction progression between the two groups (right). The time point labeled ‘0′ signifies the baseline condition prior to the onset of infection. *n* = 10 mice group^−1^. (Scale bar: 500 µm). N) Typical micro‐CT images of knee joints on the 28th day after *S. aureus* infection in vehicle and BIBN4096 treated mice (left) and the differences in tibial epiphyseal volume between the two groups (right). *n* = 5 mice group^−1^. (Scale bar: 500 µm). O) Typical H&E‐stained images of knee joints (left) and scores based on H&E staining showing differences in synovial and bone damage between the two groups on the 28th day after *S. aureus* infection (right). *n* = 8 mice group^−1^. (Scale bar: 500 µm). P) Differences in the area and thickness of the articular cartilage (AC area and AC thickness) and subchondral bone (SCB area and SCB thickness) of the tibial plateaus between the two groups on the 28th day after S. aureus infection. *n* = 8 mice group^−1^. Statistical analysis: A–E,I,K,L,N,P) Student's t tests. G) Two‐way ANOVA, Sidak post hoc test. H,M) Restricted mean survival time test. J,O) Mann‐Whitney U test. *p < 0.05, **p < 0.01, ***p < 0.001, ****p < 0.0001. ns = not significant. Mean ± SEM.

We hypothesized that CGRP release caused by *S. aureus* activation of nociceptors in the synovium may directly affect the host immune response to infection. To investigate this, we generated animals with knockout of the CGRP receptor RAMP1. Compared with *Ramp1^+/+^* littermate controls, *Ramp1^−/−^* mice exhibited reduced *S. aureus* abundance in the synovium during the acute phases of infection (**Figure** **3**A). The number of myeloid cells, represented by macrophages and neutrophils, was significantly greater in *Ramp1^−/−^* mice than in *Ramp1^+/+^* littermate controls during acute infection but declined over the course of chronic infection (Figure 3B–E). However, in the littermate controls, myeloid cells were maintained at a relatively high level due to poor infection control (Figure 3B–E). Whole‐mount immunofluorescence staining revealed that during chronic infection, GFP^+^ *S. aureus* was present in the cytoplasmic region of aggregated macrophages in the *Ramp1^+/+^* group (Figure 3F). Further sorting of these macrophages revealed that the proportion of ARG1^+^ and GFP^+^ macrophages was markedly greater in *Ramp1^+/+^* littermate controls than in *Ramp1^−/−^* mice (Figure 3G). *Ramp1^−/−^* mice exhibited reductions in both the pace and severity of joint damage during chronic infection (Figure 3H–K). Conversely, intra‐articular injection of CGRP increased *S. aureus* proliferation (Figure S4A, Supporting Information) and suppressed the number of macrophages and neutrophils within the synovium (Figure S4B,C, Supporting Information). Next, we aimed to determine whether CGRP has a role independent of RAMP1 signaling in our infection model. While the bacterial load in the joints was lower in *Ramp1^−/−^* mice than in WT control mice, CGRP did not enhance *S. aureus* infection or immunosuppressive effects in *Ramp1^−/−^* mice as it did in WT mice (Figure S4D–F, Supporting Information).

To further explore which type of immune cells plays a more significant role in the outcomes of *S. aureus* septic arthritis regulated by nociceptive nerves, we selectively deleted the RAMP1 receptor in both lymphocytes and myeloid cells. Specifically, we first bred *Cd2^cre^* mice with *Ramp1^fl/fl^* mice to generate *Cd2^ΔRamp1^* mice, in which the RAMP1 receptor is deleted in lymphocytes. We found that this deletion had no significant effect on local bacterial loads following S. aureus joint infection (Figure S4G, Supporting Information). To determine whether myeloid immune cells mediate RAMP1 signaling and influence septic arthritis, we then crossed *Ramp1^fl/fl^* mice with *Lyz2^cre^* mice to ablate the receptor in myeloid immune cells (Figure S4H, Supporting Information). Deletion of *Ramp1* in myeloid cells (*Lyz2^ΔRamp1^*) resulted in significant reductions in acute and chronic bacterial loads in the joints compared with those in control *Ramp1^fl/fl^* mice (Figure S4H, Supporting Information). Similarly, this also diminished bacterial‐induced joint damage (Figure S4I–L, Supporting Information). These results indicate that RAMP1 signaling originating from neurons inhibits the antimicrobial response of myeloid cells.

To determine the role of blocking nociceptive neuropeptides in host defense, we administered intra‐articular injections of the CGRP receptor antagonist BIBN4096 to inhibit CGRP action. Compared with vehicle‐treated mice, BIBN4096‐treated mice showed a reduced bacterial load (Figure 3L). X‐ray analysis indicated that the average time to bone destruction was significantly delayed in the BIBN4096 group compared to the vehicle group (Figure 3M), micro‐CT and HE staining revealed a lower degree of joint destruction in the treatment group (Figure 3N‐P).

### 2.4 CGRP Targets CX3CR1^+^ Lining Macrophages at the Onset of Infection

**Figure 4:**
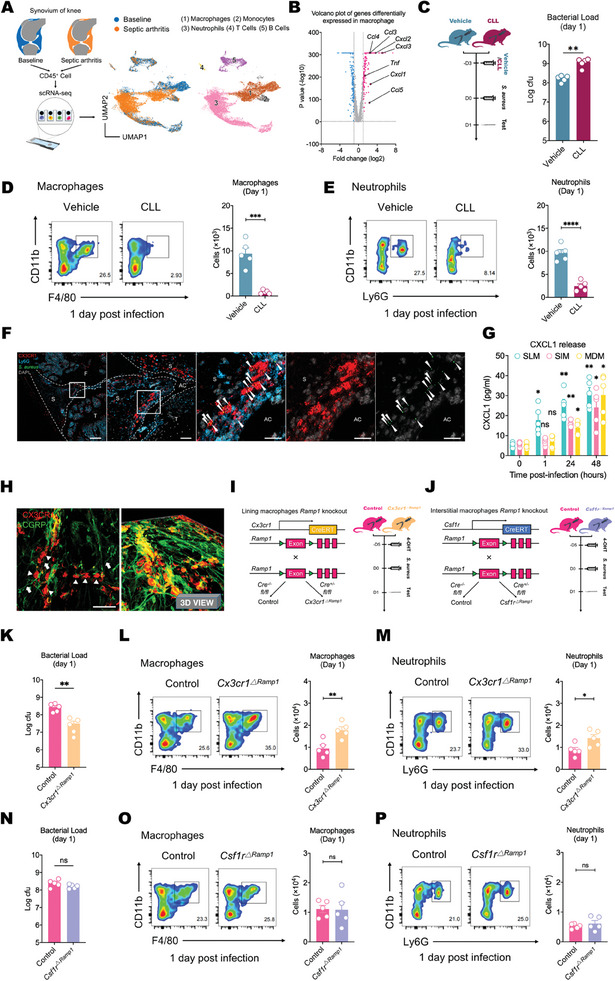
Blocking CGRP‐Ramp1 signaling in CX3CR1^+^ resident macrophages enhances initial synovial responses to *S. aureus* infection. A) UMAP visualization of CD45^+^ immune cells in the synovium of uninfected knee joints (baseline, saline injection for 24 h) and infected knee joints (1 × 10^8^ CFU of *S. aureus* infection for 24 h). B) Volcano plot showing differentially expressed genes in macrophages between infected and uninfected synovium. The red dots represent upregulated differentially expressed genes (DEGs), the blue dots represent downregulated DEGs, and the gray dots represent genes whose expression was not significantly different. *n* = 10 pooled synovium/group. C) Scheme of CLL (5 µl) pretreatment, followed by *S. aureus* (1 × 10^8^ CFU) infection (left), and the local bacterial load difference between the vehicle‐treated mice and CLL‐treated mice on the 1st day post infection (right). *n* = 5 mice group^−1^. D) Representative flow cytometry images and quantification of macrophages in the knee synovium of vehicle‐treated mice and CLL‐treated mice on the 1st day post infection. *n* = 5 mice group^−1^. E) Representative flow cytometry images and quantification of neutrophils in the knee synovium of vehicle‐treated mice and CLL‐treated mice on the 1st day post infection. *n* = 5 mice group^−1^. F) Close contact between GFP^+^ *S. aureus* and CX3CR1^+^ synovial lining macrophages was observed at 1 h after infection. The recruited ly6G^+^ neutrophils were also primarily distributed around the synovial lining (arrowheads: GFP^+^ *S. aureus*; F: femur; T: tibia; AC: articular cavity; S: synovium). (Scale bars: 300, 50, 20, 20, and 20 µm). G) ELISA of CXCL1 levels in synovial samples of CX3CR1^+^CSF1R^−^ synovial lining macrophages (SLM), CX3CR1^−^CSF1R^+^ synovial interstitial macrophages (SIM), and ly6C high CCR2^+^ monocyte‐derived macrophages (MDM) at different time points post infection. *n* = 5 mice group^−1^. H) Whole‐mount confocal images of the synovium showing the proximity of CX3CR1^+^ macrophages and CGRP^+^ nociceptive nerves in the synovium (arrows: nociceptive nerves; arrowheads: CX3CR1^+^ synovial macrophages). (Scale bar: 100 µm). I) Generation of CX3CR1^+^ synovial macrophage *Ramp1* deletion (*CX3CR1^ΔRamp1^*) mice (left). Timeline of 4‐OHT (20 µg/5 µl) administration and *S. aureus* infection (right). J) Generation of CSF1R^+^ interstitial macrophage *Ramp1* deletion (*CSF1R^ΔRamp1^*) mice (left). Timeline of 4‐OHT administration and *S. aureus* infection (right). K) Local bacterial load differences between the control group and the *CX3CR1^ΔRamp1^* group on the 1st day post infection. *n* = 5 mice group^−1^. L) Representative flow cytometry images and quantification of macrophages in the knee synovium of the control group and the *CX3CR1^ΔRamp1^* group on the 1st day post infection. *n* = 5 mice group. M) Representative flow cytometry images and quantification of neutrophils in the knee synovium of the control group and *CX3CR1^ΔRamp1^* group on the 1st day post infection. *n* = 5 mice group^−1^. N) Local bacterial load difference between the control group and *CSF1R^ΔRamp1^* group on the 1st day post infection. *n* = 5 mice group^−1^. O) Representative flow cytometry images and quantification of macrophages in the knee synovium of the control group and *CSF1R^ΔRamp1^* group on the 1st day post infection. *n* = 5 mice group^−1^. P) Representative flow cytometry images and quantification of neutrophils in the knee synovium of the control group and *CSF1R^ΔRamp1^* group on the 1st day post infection. *n* = 5 mice group^−1^. Statistical analysis: C,D,E,K–P) Student's t tests. G) Two‐way ANOVA, Dunnett post hoc test. *p < 0.05, **p < 0.01, ***p < 0.001, ****p < 0.0001. ns = not significant. Mean ± SEM.

To gain a better understanding of leukocyte responses in septic arthritis, we performed scRNA‐seq analysis on CD45^+^ immune cells collected from the joints 24 h after *S. aureus* infection and compared them to immune cells collected from uninfected controls (**Figure** **4**A). Acute infection with *S. aureus* induced significant transcriptional changes in the myeloid immune cell cluster, which consists of macrophages, monocytes, and neutrophils (Figure S5A, Supporting Information). GO enrichment analysis further highlighted the role of these cells during the acute phase of the host response to infection (Figure S5B–D, Supporting Information). Compared with those from uninfected controls, macrophages isolated from the infected synovium showed increased expression of chemotactic mediators, including *Ccl3, Ccl4, Ccl5, Cxcl1, Cxcl2* and *Cxcl3* (Figure 4B). In contrast to those in macrophages, the genes whose expression was upregulated in neutrophils and monocytes, such as *Nos2* and *Il1*, were also associated with direct pathogen cytotoxicity (Figure S5E,F, Supporting Information). Thus, intra‐articular macrophages can coordinate the antimicrobial response by recruiting immune cells such as neutrophils and monocytes that perform bactericidal functions at the onset of joint infection.

Since the macrophages involved in intra‐articular antimicrobial immunity include resident macrophages from the synovium and infiltrating monocyte‐derived macrophages, we treated the joints with clodronate‐laden liposomes (CLLs) at baseline (Figure 4C). This method targeted synovial resident macrophages without affecting neutrophils, monocytes, or lymphocytes (Figure S5G, Supporting Information). Following *S. aureus* infection, CLL treatment resulted in an overall reduction in recruited immune cells in the joints compared to those in mice treated with vehicle liposomes (Figure 4D,E; Figure S5H, Supporting Information), which led to an increased bacterial load in the joints (Figure 4C). While we cannot completely exclude the impact of CLLs on other cell types, these data suggest a critical role for resident synovial macrophages in host defense within the joints. Recent studies have identified two distinct populations of synovial resident macrophages, namely, CX3CR1^+^ lining macrophages and CSF1R^+^ interstitial macrophages, and we confirmed the existence of this structure by immunostaining (Figure S6A, Supporting Information). Lining macrophages play a crucial role in initial immune activation during inflammatory arthritis by promoting inflammatory cell infiltration, whereas interstitial macrophages differentiate to supplement lining macrophages.^[^ ^21^ ^]^ Immunofluorescence staining of the joint synovium at the onset of inflammation (1 h post infection) revealed that *S. aureus* initially interacted with the lining layer of synovial macrophages (Figure 4F), which were predominantly marked by CX3CR1.^[^ ^22^ ^]^ Ly6G^+^ che2motactic neutrophils also accumulated mainly on the lining layer of the synovium (Figure 4F). The phenomenon of neutrophil aggregation subsequently disappeared at the peak of inflammation (48 h post infection), and these Ly6G^+^ cells were ubiquitously distributed in the tissue (Figure S6B, Supporting Information). This phenomenon suggests that at the onset of infection, neutrophils respond to the chemotactic signal from CX3CR1+ macrophages. CXCL1 is the predominant cytokine that promotes neutrophil chemotaxis and activation during the early inflammatory response.^[^ ^23^ ^]^ We next assessed the expression levels of CXCL1 on the following CD11b^+^F4/80^+^ macrophage populations at 1, 24, and 48 h post infection: CX3CR1^+^CSF1R^−^ synovial lining macrophages, CSF1R^+^CX3CR1^−^ synovial interstitial macrophages and ly6C^high^CCR2^+^ monocyte‐derived macrophages.^[^ ^21^ ^]^ The results revealed that synovial lining macrophages were the first population to show an increase in inflammatory chemokines (Figure 4G).

These findings led us to hypothesize that CX3CR1^+^ synovial lining macrophages may serve as the first immune responders to invading bacteria. Synovial staining further revealed that CX3CR1^+^ macrophages in the synovial lining layer were closely associated with CGRP^+^ nerve fibers, potentially serving as the anatomical basis for initial neuroimmune regulation (Figure 4H). We generated *Cx3cr1‐creERT* mice and *Csf1r‐creERT* mice on a *Ramp1^fl/fl^* background (Figure 4I,J), and selectively depleted RAMP1 receptors in synovial lining macrophages of *Cx3cr1^△Ramp1^* mice through local injection of 4‐hydroxytamoxifen five days prior to infection (Figure 4I). Compared with those in controls, the results observed in *Cx3cr1^ΔRamp1^* mice were similar to those in *Lyz2^ΔRamp1^* mice with myeloid‐specific Ramp1 knockout, showing inhibition of *S. aureus* expansion in the synovium (Figure 4K) and increased neutrophil and macrophage infiltration after acute infection (Figure 4L,M). CSF1R is expressed only on synovial interstitial macrophages at baseline.^[^ ^21^ ^]^ We generated *Csf1r^ΔRamp1^* mice and selectively depleted RAMP1 receptors in synovial interstitial macrophages by injecting 4‐hydroxytamoxifen prior to infection (Figure 4J). During acute infection, depletion of RAMP1 receptors in CSF1R^+^ synovial interstitial macrophages did not increase leukocyte infiltration or reduce the bacterial load (Figure 4N–P). These findings indicate that synovial interstitial CSF1R^+^ macrophages are not the first responder cells in this neuroimmune pathway.

### 2.5 CGRP Targets CX3CR1^+^ and CSF1R^+^ Macrophages During Chronic Infection

**Figure 5:**
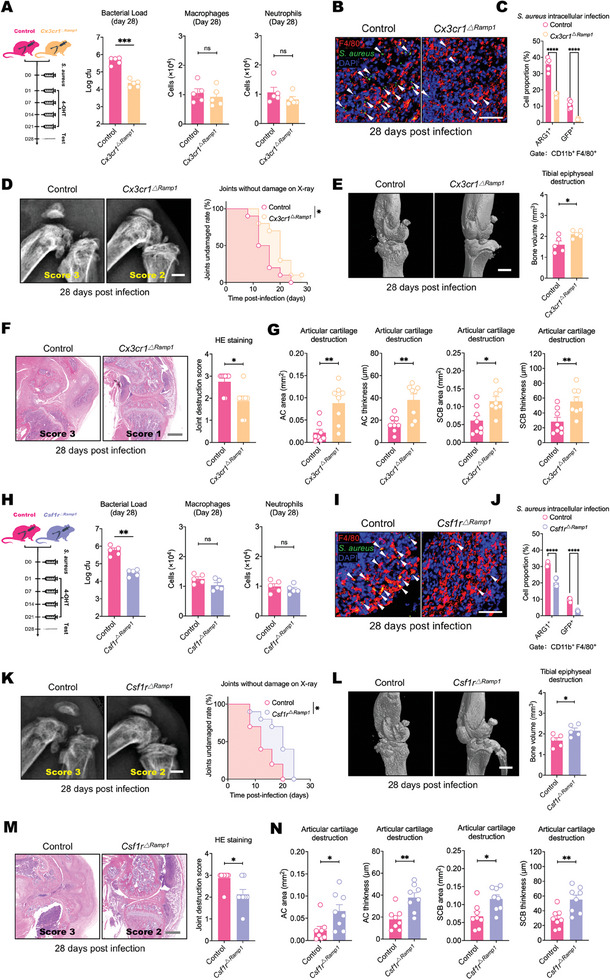
CGRP‐Ramp1 regulates CX3CR1^+^ and CSF1R^+^ macrophages during persistent infection. A) Comparison of the local bacterial load between control and *CX3CR1^ΔRamp1^* mice on the 28th day post infection (left). Flow cytometric quantification of macrophages (middle) and neutrophils (right) in the knee synovium. *n* = 5 mice group^−1^. B) Representative whole‐mount immunofluorescence images of macrophages and GFP^+^ *S. aureus* in the knee synovium of control and *CX3CR1^ΔRamp1^* mice on the 28th day after infection. (arrowheads: *S. aureus*, Scale bar: 50 µm). C) Flow cytometry quantification of anti‐inflammatory macrophages (ARG1^+^) and intracellular *S. aureus*‐harboring macrophages (GFP^+^) in the knee synovium of control and *CX3CR1^ΔRamp1^* mice on the 28th day post infection. *n* = 5 mice/group. D) Typical X‐ray images of knee joints on the 28th day after *S. aureus* (1 × 10^8^ CFU) infection in control and *CX3CR1^ΔRamp1^* mice (left) and survival curves reflecting the differences in bone destruction progression between the two groups (right). The time point labeled “0′ signifies the baseline condition prior to the onset of infection. *n* = 10 mice group^−1^. (Scale bar: 500 µm). E) Typical micro‐CT images of knee joints on the 28th day after *S. aureus* infection in control and *CX3CR1^ΔRamp1^* mice (left) and the differences in tibial epiphyseal volume between the two groups (right). *n* = 5 mice group^−1^. (Scale bar: 500 µm). F) Typical H&E‐stained images of knee joints (left) and scores based on H&E staining showing differences in synovial and bone damage between the two groups on the 28th day after *S. aureus* infection (right). *n* = 8 mice group^−1^. (Scale bar: 500 µm). G) Differences in the area and thickness of the articular cartilage (AC area and AC thickness) and subchondral bone (SCB area and SCB thickness) of the tibial plateaus between the two groups on the 28th day after S. aureus infection. *n* = 8 mice group^−1^. H) Comparison of the local bacterial load between control and *CSF1R^ΔRamp1^* mice on the 28th day post infection (left). Flow cytometric quantification of macrophages (middle) and neutrophils (right) in the knee synovium. *n* = 5 mice group^−1^. I) Representative whole‐mount immunofluorescence staining of macrophages and GFP^+^ *S. aureus* in the knee synovium of control and *CSF1R^ΔRamp1^* mice on the 28th day post infection. (arrowheads: *S. aureus*, Scale bar: 50 µm). J) Flow cytometry quantification of anti‐inflammatory macrophages (ARG1^+^) and intracellular *S. aureus*‐harboring macrophages (GFP^+^) in the knee synovium of control and *CSF1R^ΔRamp1^* mice on the 28th day post infection. *n* = 5 mice group^−1^. K) Typical X‐ray images of knee joints on the 28th day after *S. aureus* infection in control and *CSF1R^ΔRamp1^* mice (left) and survival curves reflecting the differences in bone destruction progression between the two groups (right). The time point labeled ‘0′ signifies the baseline condition prior to the onset of infection. *n* = 10 mice group^−1^. (Scale bar: 500 µm). L) Typical micro‐CT images of knee joints on the 28th day after *S. aureus* infection in control and *CSF1R^ΔRamp1^* mice (left) and the differences in tibial epiphyseal volume between the two groups (right). *n* = 5 mice group^−1^. (Scale bar: 500 µm). M) Typical H&E‐stained images of knee joints (left) and scores based on H&E staining showing differences in synovial and bone damage between the two groups on the 28th day after *S. aureus* infection (right). *n* = 8 mice group^−1^. (Scale bar: 500 µm). N) Differences in the area and thickness of the articular cartilage (AC area and AC thickness) and subchondral bone (SCB area and SCB thickness) of the tibial plateaus between the two groups on the 28th day after S. aureus infection. *n* = 8 mice group^−1^. Statistical analysis: A,E,G,H,L,N) Student”s t tests. C,J) Two‐way ANOVA, Sidak post hoc test. D,K) Restricted mean survival time test. F,M) Mann‐Whitney U test. *p < 0.05, **p < 0.01, ***p < 0.001, ****p < 0.0001. ns = not significant. Mean ± SEM.

Due to the aforementioned association between neuronal activation and CGRP release in chronic septic arthritis, we next investigated whether CGRP still plays an immunomodulatory role after infection onset. We initiated intra‐articular injections of 4‐hydroxytamoxifen, which targets the RAMP1 receptor of *Cx3cr1^ΔRamp1^* and *Csf1r^ΔRamp1^* mice, at one day post infection, followed by subsequent weekly injections. This method excludes the role of CGRP during the infection‐triggering stage. Interestingly, despite the suppression of bacterial growth, the inhibition of CGRP receptors after infection onset did not elicit enhanced recruitment of immune cells (**Figure** **5**A,H). Immunofluorescence staining revealed that intervention with the RAMP1 receptor in CX3CR1^+^ and CSF1R^+^ macrophages during chronic infection reduced the number of GFP^+^ *S. aureus* inside the macrophage cytoplasm (Figure 5B,I). Flow cytometric analysis also revealed that the percentages of ARG1^+^ and GFP^+^ macrophages were significantly lower in the two intervention groups than in the control group (Figure 5C,J). These interventions induced phenotypic changes in macrophages that potentially enhanced their bactericidal capacity, thereby ameliorating joint destruction (Figure 5D–G,K–N). These findings suggest that CGRP not only inhibits the immunochemotactic effect of CX3CR1^+^ synovial lining macrophages during the initial infection stage but also impacts the bactericidal activities of CX3CR1^+^ and CSF1R^+^ macrophages during the establishment of infection persistence.

To gain a deeper understanding of how CGRP influences the response of intra‐articular macrophages to *S. aureus*, we isolated synovial macrophages and conducted in vitro culture experiments involving CGRP treatment and bacterial infection. RNA‐seq analysis revealed that synovial macrophages stimulated with only *S. aureus* exhibited increased levels of chemotactic cytokines, including *Cxcl1, Cxcl2*, and *Ccl3* (Figure S6C, Supporting Information). However, short‐term pretreatment of synovial macrophages with CGRP resulted in the suppression of the aforementioned increase in chemotactic cytokines, accompanied by the upregulation of *Arg1* expression (Figure S6D, Supporting Information). Compared with those in the control group, prolonged exposure to CGRP led to the downregulation of cytokines (*Tnf, Nlrp3*, and *Nos2*) associated with postphagocytic killing function following *S. aureus* infection (Figure S6E, Supporting Information). In line with these findings, compared with those of the controls, the killing function of synovial macrophages treated with CGRP for a short duration was unchanged, whereas those subjected to prolonged CGRP treatment demonstrated a decrease in postphagocytic killing (Figure S6F, Supporting Information). After a 24 h infection of synovial macrophages with *S. aureus*, lysis of macrophages and assessment of the intracellular bacterial load revealed an increased number of viable bacteria in synovial macrophages treated with prolonged administration of CGRP (Figure S6G, Supporting Information). To further investigate the mechanisms underlying CGRP‐mediated modulation of synovial macrophages, we pretreated macrophages with the cAMP analog Rp‐8‐CPT‐cAMP (a PKA inhibitor, PKAi) before stimulation with CGRP and *S. aureus*. This strategy is based on the knowledge that the CGRP receptor RAMP1‐CALCRL activates Gα_s_ to increase cyclic AMP levels and activate the cAMP‐dependent protein kinase PKA to exert its effects. ELISA results revealed that short‐term CGRP treatment downregulated the expression of various chemotactic cytokines, whereas long‐term CGRP treatment downregulated the expression of cytokines related to postphagocytic bactericidal capacity. ARG1 expression was upregulated under both conditions, validating the transcriptomic sequencing results (Figure S6H,I, Supporting Information). Pretreatment with PKAi blocked the effects of CGRP under all interventions (Figure S6H,I, Supporting Information). These findings corroborate the observations of in vivo experiments, suggesting that CGRP signaling in synovial macrophages is mediated through the cAMP/PKA pathway and polarizes macrophages toward an anti‐inflammatory ARG1 phenotype but with different cytokine targets in the acute versus chronic phases.

## 3 Discussion

Septic arthritis can cause chronic pain over the long term. This study aimed to explore the mechanisms underlying this pain and whether neuroimmune signaling affects host defense. *S. aureus* was observed to activate nociceptive neurons, facilitating their invasion within the joints and thereby linking neurons to the pathogenesis of septic arthritis. In this neuroimmune axis, CGRP^+^ neurons signal to CX3CR1^+^ synovial lining macrophages through RAMP1, suppressing the recruitment of immune cells at the antimicrobial immune initiation stage. Furthermore, during subsequent chronic septic arthritis, CGRP^+^ neurons, via RAMP1, continuously modulate the postphagocytic killing function of macrophages and create a niche suitable for bacterial survival.

### 3.1 *S. Aureus* Utilizes the Repair Capacity of Nociceptor Tissue to Survive

The synovium serves as the primary functional unit within joints for sensing harmful stimuli and secreting immunoregulatory factors. It harbors nociceptive neurons and resident macrophages. This study revealed that both are intricately connected structurally. The physiological role of this neuroimmune axis in the synovial membrane remains incompletely understood; however, insights from studies by Strausbaugh et al. suggest that it may represent a strategy for limiting inflammation.^[^ ^24^ ^]^ Recent research on synovial immunity has further elucidated its role in preventing inflammatory cell infiltration in inflammatory joint diseases.^[^ ^21^ ^]^ Nociceptors limit the intensity and duration of lung and joint inflammation, thereby protecting these tissues from inflammatory damage.^[^ ^24^ ^]^ They also support wound healing in the skin.^[^ ^25^, ^26^ ^]^ CGRP is also believed to have a bone‐protective effect.^[^ ^27^ ^]^ While these data demonstrate the tissue‐protective role of CGRP, tumor cells and pathogens appear to benefit from this sensory nerve‐targeted immunomodulation.^[^ ^10^, ^11^ ^]^ Similarly, CGRP‐positive nociceptors may signal to RAMP1‐positive macrophages to promote synovial injury healing and the resolution of joint inflammation. Therefore, certain pathogens, such as *S. aureus*, may exploit the activation of this neuroimmune axis to evade immunity.

### 3.2 S. aureus Continuously Triggers Synovial Nociceptor‐Mediated CGRP Release

One issue that remains to be clarified is how nociceptive neurons mediate this immune‐modulating pain. In this study, it was observed for the first time from clinical cases that the severity of pain in patients with *S. aureus*‐induced septic arthritis was not always influenced by the extent of local inflammation. *S. aureus* strains from severe pain patients showed higher *hla* gene expression than those from mild pain patients did, which corresponded to elevated levels of tissue CGRP secretion. One possible explanation for these observations is that the pathogen can directly activate nociceptors within the joint through PFTs. To test this hypothesis, USA300 isogenic mutant strains lacking *hla* expression (*Δhla*) were generated. In vitro and in vivo experiments demonstrated that PFTs can directly activate nociceptive neurons within the knee joint, affecting pain sensitization, which is consistent with the findings of Ribeiro et al. in a study on skin infections.^[^ ^8^ ^]^ In septic arthritis, pathogens are not easily cleared from the joint, and the course of the disease is often chronic.^[^ ^28^, ^29^ ^]^ The results of this study indicate that pain sensitization caused by *S. aureus*‐induced septic arthritis can persist for up to one month and is accompanied by a sustained increase in CGRP levels in synovial tissue. However, thermal hyperalgesia and mechanical allodynia in animals infected with the *Δhla* strain were significantly alleviated during the chronic phase of septic arthritis, even though the absence of the hla gene does not completely eliminate pain. This suggests an important role of *S. aureus* PFTs in pain sensitization in chronic septic arthritis. Although previous studies have demonstrated that *S. aureus* PFTs can activate nociceptive sensory nerves within minutes to weeks following skin infection,^[^ ^20^, ^30^ ^]^ our research, which utilized a chronic septic arthritis model, revealed the sustained impact of *S. aureus* PFTs on the release of the nociceptive neuropeptide CGRP over a longer timescale (up to one month).

### 3.3 Short‐Term and Long‐Term Effects of CGRP Targeting on Macrophages

In the system of neuroimmune crosstalk within the synovium, CX3CR1^+^ macrophages are located in the lining layer of the synovium, where they play a role in protection and isolation under physiological conditions. In models of inflammatory joint diseases, they exert initial proinflammatory effects and induce leukocyte infiltration into the synovium and joint cavity.^[^ ^30^ ^]^ We found that these synovial macrophages upregulated immune chemokines immediately after *S. aureus* invasion. When we depleted the cells with CLL prior to infection, leukocyte chemotaxis was reduced during acute infection, which exacerbated the bacterial infection. This finding indicates the initiating role of these cells in the antibacterial response. Using targeted gene knockout models, we found that nociceptor‐derived CGRP suppressed the immunoregulatory effect of CX3CR1^+^ macrophages at the onset of *S. aureus* infection via RAMP1. Our in vitro experiments revealed that short‐term CGRP treatment mediated signaling that polarized synovial macrophage responses to *S. aureus* by inhibiting the production of chemokines (e.g., *Cxcl1, Cxcl2*, and *Ccl3*) and promoting the expression of immunosuppressive transcription factors (e.g., *Arg1*). Interestingly, by continuously modulating RAMP1 in CX3CR1^+^ and CSF1R^+^ macrophages after infection, we found that CGRP signal transduction influenced the immunological functions of both populations during chronic infection. Notably, these two populations represent not only tissue‐resident synovial macrophages but also macrophages derived from infiltrating monocytes after infection onset. The prolonged inhibition of CGRP receptors on heterogeneous macrophages generated effects distinct from those of acute regulation of immune cell chemotaxis, which manifested mainly as alterations in the bactericidal capacity of macrophages. This transformation may facilitate the formation of an immunosuppressive niche that allows *S. aureus* to survive and propagate in synovial tissues or even inside immune cells for a prolonged time, resulting in persistent articular destruction. It is important to note that while the activation of immune responses, including the upregulation of chemokines, is essential for fighting infections, an excess of these chemokines may also be associated with joint destruction.^[^ ^31^, ^32^ ^]^ However, based on the results of this study, compared to an excess of chemokines, the greater presence of residual *S. aureus* within the joint may have a more damaging effect on the joint in this context.

Crosstalk between neurons and macrophages plays a crucial role in host defense at other barrier sites. In the gut, sympathetic neurons maintain the anti‐inflammatory transcriptional state and tissue‐protective phenotype of muscularis macrophages by releasing norepinephrine and activating β2‐adrenergic receptors expressed by macrophages.^[^ ^33^, ^34^ ^]^ β2‐Adrenergic receptor signaling through Gαs, such as RAMP1, triggers cAMP‐mediated macrophage polarization toward a tissue‐protective phenotype.^[^ ^35^ ^]^ In the skin, the neuropeptide TAFA4 produced by GINIP^+^ sensory neurons stimulates dermal macrophages to produce IL‐10, promoting tissue healing.^[^ ^26^ ^]^ In the context of septic arthritis, we also found that this neuron‒macrophage crosstalk attenuates proinflammatory factor release as well as the bactericidal capacity of macrophages, which may aid bacteria in achieving prolonged “domestication” of the host immune system to promote their own survival.

## 4 Conclusion

Our understanding of how various synovial components interact to mediate tissue protection and host defense is still limited. The regulatory effects of nociceptors on macrophages are highly complex and depend on context. Previous studies on the skin, lungs and meninges have revealed both positive and negative effects.^[^ ^8^, ^10^, ^11^, ^26^, ^36^, ^37^, ^38^ ^]^ Currently, options such as RAMP1 antagonists, anti‐CGRP antibodies and botulinum toxin are available for conditions such as migraines.^[^ ^39^, ^40^ ^]^ In mouse models of arthritis, we found that treatment with RAMP1 antagonists ameliorated infection. These findings suggest the therapeutic potential of targeting neuroimmune signaling in chronic joint infections.

## 5 Experimental Section

### Clinical Patient Selection

The clinical case portion of this study was approved by the ethics committee of our institution (Ethics No. IACUC FJMU [2020]128). It was focused on patients diagnosed with septic arthritis caused by *S. aureus*, as confirmed by bacterial cultures of synovial fluid. All patients provided signed informed consent forms prior to their inclusion in the study. Synovial fluid samples from patients were obtained by aspiration to determine local immune cell recruitment levels (assessed by the synovial fluid white blood cell count [SF‐WBC] and polymorphonuclear percentage [SF‐PMN%]), to perform routine bacterial cultures, and to store at −80 °C for future assays. The systemic inflammatory status was evaluated by measuring the erythrocyte sedimentation rate (ESR) and C‐reactive protein (CRP) level before treatment commenced. Importantly, all clinical specimens were procured during routine diagnostic and therapeutic procedures, minimizing any additional burden on the patients.

For this study, the following inclusion and exclusion criteria was established:

Inclusion Criteria: 1) Outpatients suspected of having septic arthritis. 2) Patients who provided signed informed consent. Exclusion Criteria: 1) Patients with synovial fluid cultures indicating a non‐*S. aureus* or polymicrobial infection. 2) Patients whose isolated *S. aureus* strains did not undergo prokaryotic transcriptome sequencing. 3) Patients unable to understand or comply with the research testing procedures. 4) Patients whose clinical data were incomplete. 5) Patients who initiated antibiotic treatment prior to study commencement. 6) Patients who consumed nonsteroidal anti‐inflammatory drugs (NSAIDs) or other types of analgesics on the day of the pain perception test. We ultimately included 18 patients. The detailed process was shown in Figure S7 (Supporting Information).

### VAS Evaluation and PPT Detection of Included Patients

A questionnaire containing the VAS criteria to determine the maximum VAS score within 24 h after admission was used. The PPTs of affected joints on the same day using a force gauge (FORCE ONE FDIX, Wagner Instruments) calibrated by measuring baseline pain in areas on healthy arms was measured.^[^ ^41^ ^]^ No patients had taken NSAIDs or other analgesics on the day of testing.

The patients were divided into severe pain (VAS score>6) and mild pain (VAS score≤6) groups.^[^ ^18^ ^]^ The demographic information of the two patient groups was not significantly different (Table S1, Supporting Information).

### Sample Collection, Microbial Culture, and Strain Preservation in Septic Arthritis Patients

All knee joint aspirations and debridement surgeries were performed by the same surgical team. During surgery, synovial fluid and/or infected tissue were collected. Synovial fluids were inoculated into culture flasks (BD Biosciences, Heidelberg, Germany) for further culture. The tissue samples were placed in 2 mL Eppendorf tubes with 1 mL sterile saline and sterile steel beads and then homogenized using an automated homogenizer (Shanghai Jingxin Industrial Development Co., Ltd., China) at 60 Hz for 180 s. The homogenates were plated on blood agar (Thermo Fisher Scientific, USA) and cultured under anaerobic and aerobic conditions. Bacteria were identified using the Vitek II system (BioMérieux, USA). Once *S. aureus* was cultured, the strains were cryopreserved in 20% glycerol at −80 °C.

### RNA‐seq of S. Aureus Isolated from Septic Arthritis Patients

The preserved *S. aureus* strain was recovered at 37 °C and 5% CO_2_ for 18–22 h. Total RNA was extracted using a RNeasy Mini Kit (Qiagen). The RNA sequencing libraries were constructed following the manufacturer's instructions. Ribosomal RNA (rRNA) was removed using a rRNA removal kit. The rRNA‐depleted RNA was fragmented and reverse‐transcribed. Each sample was then amplified by PCR using P5 and P7 primers, with both primers carrying sequences that can anneal with the flow cell to perform bridge PCR and the P5/P7 primer carrying an index to enable multiplexing. The PCR products were purified, quantified, and multiplexed for sequencing on an Illumina HiSeq platform with a 2 × 150 bp paired‐end configuration. The raw sequencing data were processed by Cutadapt to remove adapters and low‐quality reads. The cleaned reads were aligned to the *S. aureus* NCTC8325 reference genome downloaded from NCBI using Bowtie2. HTSeq and DESeq2 were utilized to estimate gene expression levels and identify differentially expressed genes, respectively. Volcano plots were used to visualize the distribution of the differentially expressed genes.

### Animals

In accordance with international standards, all animal care and experiments were approved by our animal protection and use committee (IACUC FJMU 2022‐NSFC‐0368). The animals were kept in individually ventilated miniature isolation cages with free access to water and food at a temperature of 22–24 °C, a humidity of 60 ± 5%, and a 12 h light/dark cycle.

C57BL/6J mice were purchased from Beijing HFK Bioscience Co., Ltd. C57BL/6JSmoc‐*Trpv1^em1(Myc‐IRES‐Cre)Smoc^* (NM‐KI‐200139), C57BL/6JSmoc‐*Ramp1^em1Smoc^* (NM‐KO‐201764) and C57BL/6Smoc‐*Cd2^em(IRES‐Cre)1Smoc^* (NM‐KI‐225032) mice were purchased from Shanghai Biomodel Organism Science and Technology Development Co., Ltd. C57BL/6JGpt‐*Csf* *1r^em1Cin(P2A‐CreERT2)^*/Gpt (T006204) and B6/JGpt‐*Ramp1^em1Cflox^*/Gpt (T0096^44^) mice were purchased from GemPharmatech (Nanjing, China). C57BL/6‐*Gt(ROSA)26Sor^1(HBEGF)Awai^*/J (JAX 0 07900), B6.129P2‐*Lyz2^1(cre)Ifo^*/J (JAX 0 04781) and B6.129P2(C)‐*Cx3cr1^2.1(cre/ERT2)Jung^*/J (JAX 02 0940) mice were purchased from Jackson Laboratories.

C57BL/6JSmoc‐*Trpv1* ^em1(Myc‐IRES‐Cre)Smoc^ (NM‐KI‐200139) heterozygous mice were bred with C57BL/6‐Gt(ROSA)26Sor^1(HBEGF)Awai/J^ (JAX 0 07900) homozygous mice to generate *Trpv1‐Cre/*DTR (*Trpv1‐Cre* ^+/−^‐DTR^+/−^) mice as well as littermate control mice (*Trpv1‐Cre* ^−/−^‐DTR^+/−^). C57BL/6JSmoc‐*Ramp1^em1Smoc^* (NM‐KO‐201764) heterozygous mice were bred together to produce both RAMP1‐knockout (*Ramp1^−/−^*) mice and littermate control (*Ramp1* ^+/+^) mice. For the conditional knockout experiments, we generated *Lyz2^ΔRamp1^* (B6.129P2‐*Lyz2^1(cre)Ifo^*/J*^+/−^*;*Ramp1^fl/fl^*), *CD2^ΔRamp1^* (C57BL/6Smoc‐*Cd2^em(IRES‐Cre)1Smoc+/−^*;*Ramp1^fl/fl^*), *Cx3cr1^ΔRamp1^* (B6.129P2(C)‐*Cx3cr1^2.1(cre/ERT2)Jung^*/J^+/−^;*Ramp1^fl/fl^*), and *Csf1r^ΔRamp1^* (C57BL/6JGpt‐*Csf* *1r^em1Cin(P2A‐CreERT2)^*/Gpt^+/−^;*Ramp1^fl/fl^*) mice. These mice were then bred with *Ramp1^fl/fl^* (T009644) mice to generate mice with specific depletion of RAMP1 in myeloid cells (*Lyz2^ΔRamp1^*), lymphocytes (*CD2^ΔRamp1^*), synovial lining macrophages (*Cx3cr1^ΔRamp1^*), synovial interstitial macrophages (*Csf1r^ΔRamp1^*), and their respective control littermates (cre^−/−^; *Ramp1^fl/fl^*). For all experiments conducted in this study, both male and female mice ranging from 8–14 weeks of age were used, with age matching ensured between groups.

### Bacterial Strains and Cultures

The MRSA strain USA300 was used in this study. *S. aureus* with *hla* gene deletion (*∆hla*) was generated as previously described.^[^ ^42^, ^43^ ^]^ GFP^+^ *S. aureus* was purchased from Forhigh Biotech (Hangzhou, China, cat. no. BSM1085). The recombinant plasmid *pCM: hla* was electroporated into *∆hla S. aureus* to obtain the complemented strain *pCM: hla*+*∆hla S. aureus*. The bacterial stocks were stored at −80 °C in glycerol. The stocks were recovered on LB agar (Sangon Biotech) at 37 °C and 5% CO2 for 18–22 h before used. Bacterial suspensions were prepared in sterile saline and quantified by measuring the optical density.

### Construction of an S. Aureus Septic Arthritis Mouse Model

The mice were anesthetized with 2%–3% isoflurane, and the lower limbs were shaved. The right knee joint was disinfected with 75% ethanol, and then a longitudinal incision of ≈1 mm was carefully made through the skin overlying the patellar tendon to expose it. A Hamilton microsyringe attached to a 34‐gauge needle was used to penetrate the knee joint cavity through the midpoint of the patellar tendon. A volume of 1 µL of bacterial suspension containing 1 × 10^8^ CFU of *S. aureus* was injected into the joint cavity, after which the incision was sutured with sterile silk and disinfected again with 75% ethanol. The aseptic technique was maintained throughout to prevent contamination. We constructed the septic arthritis mouse model using various strains of *S. aureus*, all following the same methodology.

### Bacterial Load Measurement

To determine the bacterial load in the tissues, after euthanasia, the periarticular tissues were promptly harvested and transferred into sterile Eppendorf tubes. The tissues were homogenized in sterile saline using an automated homogenizer (Jingxin Industrial Development Co., Shanghai, China) at 60 Hz for 180 s. Tissue homogenates were serially diluted and plated on LB agar (Sangon Biotech) overnight at 37 °C and 5% CO_2_.

For in vitro cell infection experiments, extracellular bacteria were directly assessed by diluting and plating the cell culture supernatant on LB agar. Intracellular bacteria were assessed by lysing the cells with 0.1% Triton for 5 min to release intracellular bacteria after killing extracellular bacteria via 20 µg mL^−1^ lysostaphin (Sigma‒Aldrich) treatment for 30 min and plating them on LB agar overnight at 37 °C and 5% CO_2_. The bacterial load was determined by counting the number of colony forming units (CFUs).

### Assessment of Mechanical and Thermal Hyperalgesia

Mechanical hyperalgesia was examined using von Frey filaments and the up–down method.^[^ ^42^ ^]^ The 50% paw withdrawal threshold (PWT) was calculated using the following formula: 10^[Xf + kδ]^/10 000, where Xf was the value of the final von Frey filament (in log units), k is from the response pattern, and δ is the mean difference between stimuli (in log units).^[^ ^44^ ^]^ Thermal hyperalgesia was assessed using a Hargreaves apparatus (Ugo Basile, Italy).^[^ ^45^ ^]^ The mice were placed on the glass of the machine and acclimated until exploratory behavior ceased. Radiant heat (25% of the maximum intensity) was applied to the plantar surface. Paw withdrawal latency was defined as the time for the mouse to withdraw its paw from the thermal stimulus. Three measurements were taken for each mouse at each time point and averaged. A 30 s cutoff was set to prevent tissue damage.

### Histological Staining and Histological Scoring

After euthanasia, the mouse hearts were perfused with 50 mL of PBS followed by 50 mL of 4% paraformaldehyde (PFA) for fixation. The extracted knee joints were immersed in 4% PFA for 24 h. After dehydration in 30% sucrose, the tissues were embedded in paraffin blocks and sectioned into 5 µm sagittal slides using a microtome (Thermo, Germany). Prior to staining, the paraffin‐embedded sections were dewaxed and rehydrated. For H&E staining, the sections were stained with hematoxylin solution for 5 min, rinsed in tap water, differentiated in acid ethanol, blued in bicarbonate solution, and rinsed in running water. Then, the sections were dehydrated through gradients of 85% and 95% ethanol, stained with eosin solution for 5 min, and dehydrated in absolute ethanol I, II, and III for 5 min each. Finally, the sections were cleared in xylene and sealed with neutral gum. Microscopic examination, imaging and analysis were subsequently performed. The degree of synovitis and bone destruction on H&E‐stained sections was graded on a 0–3 scale as follows: grade 0, no signs of inflammation or bone erosion; grade 1, mild synovial hyperplasia and bone erosion; grade 2, moderate synovial hyperplasia and bone erosion; and grade 3, marked synovial hyperplasia and severe bone erosion.^[^ ^46^ ^]^ Additionally, it was also measured the area and thickness of the articular cartilage (AC area and AC thickness) and the subchondral bone (SCB area and SCB thickness) of the tibial plateaus, assessed with ImageJ software (NIH, Bethesda, MD, USA).

### X‐Ray Examination and Radiological Scoring

X‐ray radiography of the knee joint was performed before and after knee infection. Specifically, the mice were placed in the prone position and imaged using animal X‐ray equipment to evaluate the degree of bone destruction in the knee joint. The extent of articular destruction on X‐ray images was graded on a 0–3 scale as follows: grade 0, no detectable signs of bone or cartilage erosion; grade 1, mild osteophyte formation and erosion of the articular surface; grade 2, moderate osteophyte formation and erosion of the articular surface, which may be accompanied by subluxation; and grade 3, massive osteophyte formation and severe destruction of the joint integrity, which is often accompanied by subluxation.

### Micro‐Computed Tomography (Micro‐CT) Analysis

The knees of mice from different groups were harvested and fixed in 4% paraformaldehyde at room temperature for one day. Subsequently, the tissues were washed three times with PBS and stored in 70% ethanol for micro‐CT processing. To assess joint destruction, specifically using tibial epiphyseal destruction as a representative measure, micro‐CT imaging was performed with a Skyscan 1172 scanner (Bruker, Kontich, Belgium). Images from each group were reconstructed at identical thresholds to enable 3D structural rendering of each joint. Bone volume analysis was conducted on the tibial epiphysis (distal end of the tibial growth plate).

### Transmission Electron Microscopy (TEM)

After perfusion, a 1 mm^3^ synovium sample was postfixed in EM fixative (2.5% glutaraldehyde and 2% paraformaldehyde in 0.1 M PB, pH 7.4), osmicated in 2% osmic acid, dehydrated in a graded alcohol series (30%–95%), and embedded in pure Epon at 60 °C for 72 h. Ultrathin sections (80 nm) were cut using an ultramicrotome, collected on 200 mesh copper grids, and stained with 3% lead citrate and 8% uranyl acetate solutions. Ultrastructural analysis was conducted via electron microscopy, and the resulting data were digitalized.

### Local Joint Ablation of Nociceptors Using RTX or Diphtheria Toxin (DTX)

To selectively ablate the intra‐articular nociceptors, WT mice received intra‐articular injections of resiniferatoxin (RTX, 100 ng/5 µL, CFWLABS, Cat# CFW‐AN125100U) or vehicle 3 weeks before infection. For *Trpv1‐Cre* ^+/−^‐DTR^+/−^ mice, DTX (10 ng/5 µL, ITI BioChem, ITI011691) was administered intra‐articularly to ablate local TRPV1*^+^* nociceptors 3 weeks before infection, and *Trpv1‐Cre* ^−/−^‐DTR^+/−^ littermates were used as controls.

### Local Joint Administration of 4‐Hydroxytamoxifen, CGRP and BIBN4096

To modulate neuropeptide levels, CGRP (2 µg/5 µL, MedChemExpress, Cat. No. HY‐P0203A) and the vehicle control were preinjected intra‐articularly into the knee joint cavities of the mice 2 h prior to infection. To block neuropeptide, CGRP receptor antagonist BIBN4096 (5 µg/5 µL, MedChemExpress, Cat. No. HY‐10095), along with vehicle controls, were administered via intra‐articular injection into the knee joint 2 h before infection, followed by weekly injections thereafter.^[^ ^47^ ^]^ For inducible activation of Cre recombinase, (Z)‐4‐hydroxytamoxifen (4‐OHT, 20 µg/5 µL, Sigma‒Aldrich, Cat. No. H7904) was injected into the knee joint.^[^ ^21^ ^]^ For acute septic arthritis studies, 4‐OHT injection preceded infection by 5 days, whereas for chronic studies, it was given 1 day after infection and weekly thereafter. This enabled temporal control over Cre‐lox recombination relative to septic arthritis induction.

### Immune Cell Depletion

For depletion of synovial macrophages, 5 µl of CLLs (Yeasen Biotechnology [Shanghai] Co., Ltd., Cat# 40337ES10) was delivered intra‐articularly into the mice 3 days before infection. The control mice received an equal volume of empty control liposomes (Yeasen Biotechnology [Shanghai] Co., Ltd., Cat# 40338ES10).

### Fluorogold (FG) Retrograde Tracing

Ten‐week‐old C57BL/6J mice were anesthetized with isoflurane, and 2 µl of 2% FG (FLUOROCHROME, LLC) was injected through the patellar ligament with a 33G needle. Three days later, L1 to L6 DRGs were collected for immunofluorescence staining.

### Isolation and Culture of DRG Neurons

DRG neurons were isolated and cultured as described previously.^[^ ^48^ ^]^ Briefly, DRGs were harvested from 6–8‐week‐old mice after euthanasia and disinfected in 75% ethanol for 10 min. Subsequent procedures were performed under sterile conditions. The intact vertebral columns were removed, and the lumbar vertebrae (L3‐L5) were isolated using sterilized microscissors and microforceps under a dissection microscope. The vertebral canals were opened with the tip of microscissors along the midline. The residual spinal cord tissues were carefully removed with microforceps and microscissors. L3‐L5 DRGs were then carefully dissected through the intervertebral foramen. The extracted DRGs were transferred to Neurobasal A medium (Thermo Fisher) and finely minced using microscissors. The minced tissues were centrifuged at 1000 rpm for 3 min, and the supernatant was discarded. The minced tissue was digested in 2 mL of mixed enzymatic solution with collagenase A (1.25 mg mL^−1^, Sigma‒Aldrich) and dispase II (2.5 mg mL^−1^, Sigma‒Aldrich) in an incubator with shaking (37 °C, 150 rpm) for 15 min. After centrifugation at 1000 rpm for 5 min at 4 °C, the supernatant was removed. The tissue was resuspended in 1 mL of Neurobasal A medium supplemented with 10% FBS to terminate digestion and then incubated with 150 U mL^−1^ DNAse I (TransGen Biotech). To dissociate the DRG cells into a single‐cell suspension, mechanical trituration of the DRG mixture was performed by sequentially aspirating and ejecting through 18G, 21G and 26G sterile needles with decreasing diameters. The cell suspension was filtered through a 70 µm cell strainer and centrifuged at 1000 rpm for 5 min at 4 °C. The dissociated DRG cells were resuspended in DRG culture medium consisting of Neurobasal A medium supplemented with 50 ng mL^−1^ nerve growth factor (NGF, Thermo Fisher) and 10 µM cytosine arabinoside (Sigma) for cell counting and plating on 10 µg mL^−1^ laminin‐coated plates.

### DRG Neuron Calcium Imaging

The method for obtaining DRG neurons is described in the section “*Isolation and Culture of DRG Neurons*”. DRG neurons were cultured on 35 mm glass‐bottom dishes for one day prior to the experiment. The cultured DRG neurons were then loaded with 5 µM Fura‐2AM (Thermo Fisher) at 37 °C for 30 min. After washing with PBS to remove excess dye, the neurons were incubated in Ringer's solution. Intracellular calcium was monitored using an IX83 (Olympus) inverted fluorescence microscope equipped with an opti‐MOS sCMOS (QImaging) camera. During imaging, bacterial supernatants from *S. aureus* (1 × 10^7^, 1 × 10^8^, or 1 × 10^9^ CFU), *∆hla S. aureus* (1 × 10^9^ CFU), *pCM:hla+∆hla S. aureus* (1 × 10^9^ CFU), or Hla (Sigma‒Aldrich, 1 to 100 µg ml^−1^), as well as capsaicin (1 µM) and KCl (40 mM), were administered to the neurons via a Multichannel Systems PPS2 (Harvard Bioscience) perfusion system. Fura‐2AM was excited using alternating ultraviolet light at 340 and 380 nm wavelengths, targeting both calcium‐bound or free form. Image acquisition and analysis were performed using cellSens software. The relative changes in intracellular calcium levels were indicated by the ratio of the emission intensity after excitation with ultraviolet light at 340 nm to that at 380 nm wavelengths (F340/F380). An increase of 10% or greater compared with baseline in F340/F380 was defined as evidence of elevated intracellular calcium.

### Isolation and Culture of Synovial Macrophages

Synovial tissues were surgically isolated from the knee joints of 6‐ to 8‐week‐old mice as previously described.^[^ ^49^ ^]^ The tissues were minced thoroughly and digested in 1 mL of RPMI 1640 medium containing 1.25 mg mL^−1^ collagenase A and 2.5 mg mL^−1^ dispase II at 37 °C for 1 h with shaking. The cell suspension was passed through a 70 µm cell strainer to obtain single cells. The cells were seeded onto type I collagen‐coated dishes in RPMI 1640 medium (Thermo Fisher Scientific) supplemented with 10% FBS and cultured at 37 °C for 1 h to allow fibroblast attachment. The nonadherent cells were transferred to noncoated dishes. After 1 day of culture at 37 °C, the medium was removed to eliminate nonadherent lymphocytes. The cells were further cultured in RPMI 1640 with 10% FBS for 1–2 weeks with regular medium changes. During this period, 0.05% trypsin digestion for 3 min was performed to selectively remove residual fibroblasts.^[^ ^50^ ^]^

### RNA‐seq of Synovial Macrophages

Synovial macrophages were cultured as described in the *“Isolation and culture of synovial macrophages”* section. The macrophages were divided into the following four groups: 1) a vehicle control group, 2) an *S. aureus* infection (MOI = 100) group, 3) a group pretreated with CGRP for 2 h and infected with *S. aureus* (MOI = 100), and 4) a group pretreated with CGRP for 72 h and then infected with *S. aureus* (MOI = 100). Total RNA was extracted using TRIzol reagent and reverse‐transcribed into cDNA libraries. The cDNA libraries were sequenced on the Illumina sequencing platform. The raw image data acquired through sequencing were transformed into sequence data via base calling. Subsequently, fastp was used for quality control. The RMA algorithm performs background correction, log2 transformation, and normalization processing to generate processed data with a low background signal. Gene expression trend analysis was conducted using the OmicShare tools platform (www.omicshare.com/tools) with Short Time‐series Expression Miner (STEM) software. Volcano plot analysis using ggplot2 was performed to visualize the differential gene expression between the comparison groups.

### scRNA‐seq of the Synovium

Before sampling, mice in the infection group received an intra‐articular injection of 1 µL of bacterial suspension containing 1 × 10^8^ CFU of *S. aureus*, while the mice in the control group were injected with an equivalent volume of sterile saline, 24 h prior to synovial tissues collection. The specific procedures for this operation were described in the section “*Construction of an S. aureus Septic Arthritis Mouse Model*”.

The synovial tissues were stored and washed with Hanks’ balanced salt solution (HBSS) three times, minced into small pieces, and then digested with 3 mL of sCelLive Tissue Dissociation Solution (Singleron) from a Singleron PythoN Tissue Dissociation System at 37 °C for 15 min. The cell suspension was collected and filtered through a 40‐micron sterile strainer. Afterward, GEXSCOPE red blood cell lysis buffer (RCLB, Singleron) was added, and the mixture (cell:RCLB = 1:2 (volume ratio)) was incubated at room temperature for 5–8 min to remove red blood cells. The mixture was then centrifuged at 300 × g and 4 °C for 5 min, the supernatant was removed, and the pellet was suspended gently in PBS. The samples were subsequently stained with Trypan blue, and cell viability was evaluated microscopically. After the sample quality was tested, synovium samples containing synovial immune cells (CD45‐enriched) or synovial nonimmune cells (CD45‐depleted) were obtained with CD45 Microbeads (Miletnyi Biotec) and used for microwell‐based scRNA‐seq (Singleron).

Single‐cell suspensions (2 × 10^5^ cells mL^−1^) in PBS (HyClone) were loaded onto a microwell chip using the Singleron Matrix single‐cell processing system. Barcoding beads were subsequently collected from the microwell chip, after which the mRNA captured by the barcoding beads was reverse transcribed to obtain cDNA, and PCR amplification was performed. The amplified cDNA was then fragmented and ligated with sequencing adapters. The scRNA‐seq library preparations were constructed according to the GEXSCOPE single‐cell RNA library kit (Singleron) protocol and sequenced (Illumina NovaSeq 6000 System).

After sequencing, the raw reads were processed to generate gene expression profiles using CeleScope v1.5.2 (Singleron Biotechnologies) with the default parameters. Briefly, barcodes and UMIs were extracted from the R1 reads and corrected. Adapter sequences and poly A tails were trimmed from the R2 reads, and the trimmed R2 reads were aligned against the GRCm38 (10 mm) transcriptome using STAR (v2.6.1b). Uniquely mapped reads were then assigned to exons with FeatureCounts (v2.0.1). Successfully assigned reads with the same cell barcode, UMI and gene were grouped together to generate the gene expression matrix for further analysis.

After primary analysis of the raw read data, Scanpy v1.8.2 was used for quality control, dimensionality reduction and clustering in Python 3.7. For each sample dataset, the expression matrix by the following criteria was filtered: 1) cells with a gene count less than 200 or with a top 2% gene count were excluded, 2) cells with a top 2% UMI count were excluded, 3) cells with a mitochondrial content > 20% were excluded, and 4) genes expressed in fewer than 5 cells were excluded. After filtering, 24092 cells were retained for the downstream analyses, with an average of 1340.8 genes and 4267.8 UMIs per cell. The raw count matrix was normalized by total counts per cell and logarithmically transformed into a normalized data matrix. The top 2000 variable genes were selected by setting the flavor to “seurat”. Principal component analysis (PCA) was performed on the scaled variable gene matrix, and the top 20 principal components were used for clustering and dimensionality reduction. The cells were separated into different clusters via the Louvain algorithm, and the resolution parameter was set to 1.2. The cell clusters were visualized via uniform manifold approximation and projection (UMAP).

The cell type identification of each cluster was determined according to the expression of canonical markers from the reference database SynEcoSys (Singleron Biotechnology), which contains collections of canonical cell type markers for single cell seq data from CellMakerDB, PanglaoDB and recently published literature. The canonical markers and their corresponding cell types were represented by bubble plots. The genes of interest were also presented in a bubble plot. Differential gene expression analysis was performed on the sequencing datasets using Seurat v3.1.2 FindMarkers on the basis of the Wilcox likelihood ratio test with default parameters. The list of differentially expressed genes was used in pathway enrichment analysis and to create volcano plots. Gene Ontology (GO) terms in the biological process category with *p* < 0.05 were considered significant.

### Immunofluorescence Staining

The mice were perfused with 50 mL of PBS followed by 50 mL of 4% PFA for fixation. Knee joints were extracted, fixed in 4% PFA overnight at 4 °C, and decalcified in 14% EDTA for 2 weeks. The decalcified knee joints were embedded in OCT compound (Sakura Finetek, 4583) and cryosectioned at a thickness of 20 µm. The cryosections were permeabilized with 0.3% Triton X‐100 and blocked with 5% goat serum for 2 h at room temperature. The sections were incubated overnight at 4 °C with the following primary antibodies: mouse anti‐TRPV1 (Abcam, ab203103, 1:500), rabbit anti‐CGRP (Cell Signaling Technology, 14 959, 1:400), mouse anti‐CGRP (Abcam, ab81887, 1:100), rabbit anti‐TUBB3 (Abcam, ab18207, 1:500), mouse anti‐F4/80 (Santa Cruz, sc‐377009, 1:200), rabbit anti‐Ly6G (Servicebio, GB11229‐100, 1:200), mouse anti‐CX3CR1 (Santa Cruz, sc‐377227, 1:200), rabbit anti‐CX3CR1 (Invitrogen, 702 321, 1:250), and mouse anti‐CSF1R (Santa Cruz, sc‐46662, 1:200). After rinsing with PBS (5 × 5 min), the sections were incubated for 2 h at room temperature with the following secondary antibodies: goat anti‐rabbit IgG Alexa Fluor 488 (Abcam, ab150077, 1:500), goat anti‐mouse IgG Alexa Fluor 488 (Abcam, ab150113, 1:500), goat anti‐rabbit IgG Alexa Fluor 555 (Abcam, ab150078, 1:500), goat anti‐mouse IgG Alexa Fluor 555 (Abcam, ab150114, 1:500), goat anti‐rabbit IgG Alexa Fluor 594 (Abcam, ab150080, 1:500), goat anti‐mouse IgG Alexa Fluor 594 (Abcam, ab150116, 1:500), goat anti‐rabbit IgG Alexa Fluor 647 (Abcam, ab150079, 1:500), and goat anti‐mouse IgG Alexa Fluor 647 (Abcam, ab150115, 1:500). After rinsing with PBS (5 × 5 min), the nuclei were counterstained with DAPI. To enable multiplexed immunofluorescence staining using primary antibodies from the same host, a Multiple‐fluorescence Immunohistochemical Mouse/Rabbit Kit (Immunoway, RS0035) was used according to the manufacturer's instructions. For whole‐mount immunofluorescence staining, synovial tissues were surgically isolated from knee joints as previously described.^[^ ^47^ ^]^ The free‐floating synovial samples were incubated in 24‐well plates and stained using a similar immunofluorescence protocol. Fluorescence imaging was performed using a confocal microscope (Zeiss).

### Flow Cytometry and Fluorescence‐Activated Cell Sorting

After euthanasia, mouse knee synovial tissues were harvested, minced thoroughly, and digested in 1 mL of RPMI 1640 medium containing 1.25 mg mL^−1^ collagenase A and 2.5 mg mL^−1^ dispase II with shaking at 37 °C for 1 h. The cell suspension was filtered through a 70 µm cell strainer to obtain a single‐cell suspension. Cells were stained with BD Horizon Fixable Viability Stain 700 (BD Biosciences, 564 997, 1:1000) for 15 min at 4 °C, protected from light. After washing, cells were incubated with FcR blockers (BD Biosciences, 553 142, 1:100) on ice for 10 min and then stained with the following antibodies on ice for 30 min: anti‐mouse CD45, BV421 (BioLegend, 103 134, 1:100); anti‐mouse CD11b, PE‐Cy5 (BioLegend, 101 210, 1:100); anti‐mouse F4/80, APC (eBioscience, 17‐4801‐80, 1:100); anti‐mouse Ly6G, BV510 (BD, 740 157, 1:100); anti‐mouse Ly6C, FITC (BD, 553 104, 1:100); anti‐mouse CD3, APC‐Cy7 (BD, 557 596, 1:100); anti‐mouse CD19, PE (BD, 557 399, 1:100); anti‐mouse CD4, APC (BioLegend, 100 412, 1:100); anti‐mouse CD8, FITC (BioLegend, 100 706, 1:100); anti‐mouse γδ TCR, BV510 (BioLegend, 118 131, 1:100); anti‐mouse ARG1, PE (eBioscience, 12‐3697‐82, 1:100); anti‐mouse CX3CR1, BV510 (BioLegend, 149 025, 1:1000); anti‐mouse CSF1R, APC‐Cy7 (BioLegend, 135 532, 1:100); and anti‐mouse CCR2, PE (BioLegend, 150 610, 1:100). After washing, the cells were resuspended in HBSS. Flow cytometry was performed with a BeckMan CytoFLEX. The cells were sorted with a BD FACSAria III. The data were analyzed with FlowJo software (v10).

### Quantitative RT‒PCR (qRT–PCR)

Total RNA was extracted using TRIzol reagent (Thermo Fisher). cDNA was synthesized from total RNA using TransScript Uni All‐in‐One First‐Strand cDNA Synthesis SuperMix (TransGen Biotech). qRT‒PCR was performed on an ABI 7500 real‐time PCR system using PerfectStart Green qPCR SuperMix (TransGen Biotech). All reactions were run in triplicate. The mRNA levels were normalized to those of 16S rRNA and quantified using the 2 ^−∆∆Ct^ method. The following primer sequences (5′‐to‐3′) were used: *hla* forward: CTGATTACTATCCAAGAAATTCGATTG, *hla* reverse CTTTCCAGCCTACTTTTTTATCAGT; *pvl* forward: GTGCCAGACAATGAATTACCC, *pvl* reverse TTCATGAGTTTTCCAGTTCACTTC; *lukD* forward: TGAAAAAGGTTCAAAGTTGATACGAGT, *lukD* reverse TGTATTCGATAGCAAAAGCAGTGCA; *hlγ* forward: TGGCTCATTCAACTACTC, *hlγ* reverse TTCTATCAACGGCTAAAC; *16S rRNA* forward: TACACACCGCCCGTCACA, *16S rRNA* reverse CTTCGACGGCTAGCTCCAAA.

### Enzyme‐Linked Immunosorbent Assay (ELISA)

Mouse tissues were frozen in liquid nitrogen, ground thoroughly and lysed in RIPA tissue lysis buffer (Yoche Biotech) containing 1% proteinase inhibitor and 1% phosphatase inhibitor (Yoche Biotech) on ice for 30 min. The lysates were centrifuged at 4 °C, and the supernatants were collected. The conditioned medium of cultured DRG neurons was also harvested after stimulation with *S. aureus* or Hla. CGRP levels in mouse samples were quantified using a mouse CGRP ELISA kit (Cayman Chemical, Cat# 589 001). The synovial fluid of patients was centrifuged to remove cells and debris, and the supernatant was used to quantify CGRP levels by ELISA. CGRP levels in human samples were quantified using a human CGRP ELISA kit (Mlbio, Cat# ml026014).

For cytokine measurement in primary synovial macrophages, cells were obtained as described in the “*Isolation and culture of synovial macrophages*” section. The macrophages were divided into six groups and subjected to different treatments: 1) the vehicle control group; 2) the *S. aureus* infection (MOI = 100) group; 3) the group pretreated with CGRP (100 nM) for 2 h and then infected with *S. aureus* infection (MOI = 100); 4) the group pretreated with PKAi (10 µM) for 2 h, pretreated with CGRP for 2 h and then infected with *S. aureus* (MOI = 100); 5) the group pretreated with CGRP for 72 h and then infected with *S. aureus* (MOI = 100); and 6) the group pretreated with PKAi for 2 h, pretreated with CGRP for 72 h, and then infected with *S. aureus* (MOI = 100). After treatment, the cells were lysed with RIPA buffer, and the protein concentration was quantified with a Pierce Rapid Gold BCA Protein Assay Kit (Thermo Scientific, A53227). For assessment of the levels of CXCL1 (Abcam, USA; Mouse # ab216951), CXCL2 (Abcam, USA; Mouse # ab204517) and CCL3 (Abcam, USA; Mouse # ab200017), cell culture supernatants were collected after treatment. Cell lysates were harvested and utilized for the measurement of the expression of ARG1 (Abcam, USA; Mouse # ab269541), NOS2 (Abcam, USA; Mouse # ab253219) and NLRP3 (Abcam, USA; Mouse # ab279417). ELISAs were performed following the manufacturers’ protocols.

### Statistical Analysis

Statistical analyses were performed using SPSS, R and GraphPad Prism software. The data were expressed as the mean ± standard error of the mean (SEM). The Spearman rank correlation test was used to analyze the correlation between two groups of data. Unpaired Student's t test was used to compare single variables with a normal distribution between two groups. One‐way ANOVA and two‐way ANOVA were used for comparisons of more than two groups or two conditions. The Mann‐Whitney U test was employed for comparisons between two groups of ordinal data, while the Kruskal‐Wallis H test was used to compare differences among more than two groups. Survival curves were compared using the restricted mean survival time (RMST) test. Differences with *p* < 0.05 were considered statistically significant.

## Conflict of Interest

The authors declare no conflict of interest.

## Supporting information



Supporting Information

## Data Availability

The data that support the findings of this study are available from the corresponding author upon reasonable request.
