# Supplementary material for: Staphylococcus Aureus Tames Nociceptive Neurons to Suppress Synovial Macrophage Responses for Sustained Infection in Septic Arthritis
Source: Adv Sci (Weinh). 2025 Feb 17;12(14):2409251. doi: 10.1002/advs.202409251 (PMC11984863; doi:10.1002/advs.202409251)
Supplement: Supplementary file 1 — Supporting Information [file ADVS-12-2409251-s001.docx]

***Staphylococcus aureus* Tames Nociceptive Neurons to Suppress Synovial Macrophage Responses for Sustained Infection in Septic Arthritis**

*Xinyu Fang, Yang Chen, Haiqi Ding, Changyu Huang, Hongxin Hu, Chaofan Zhang, Yunzhi Lin, Qijin Wang, Xueni Hu, Yiming Lin, Yongfa Chen, Nanxin Zhang, Xuhui Yuan, Ying Huang, Wenbo Li, Susheng Niu, Jianhua Lin, Bin Yang*, Tifei Yuan*and Wenming Zhang**


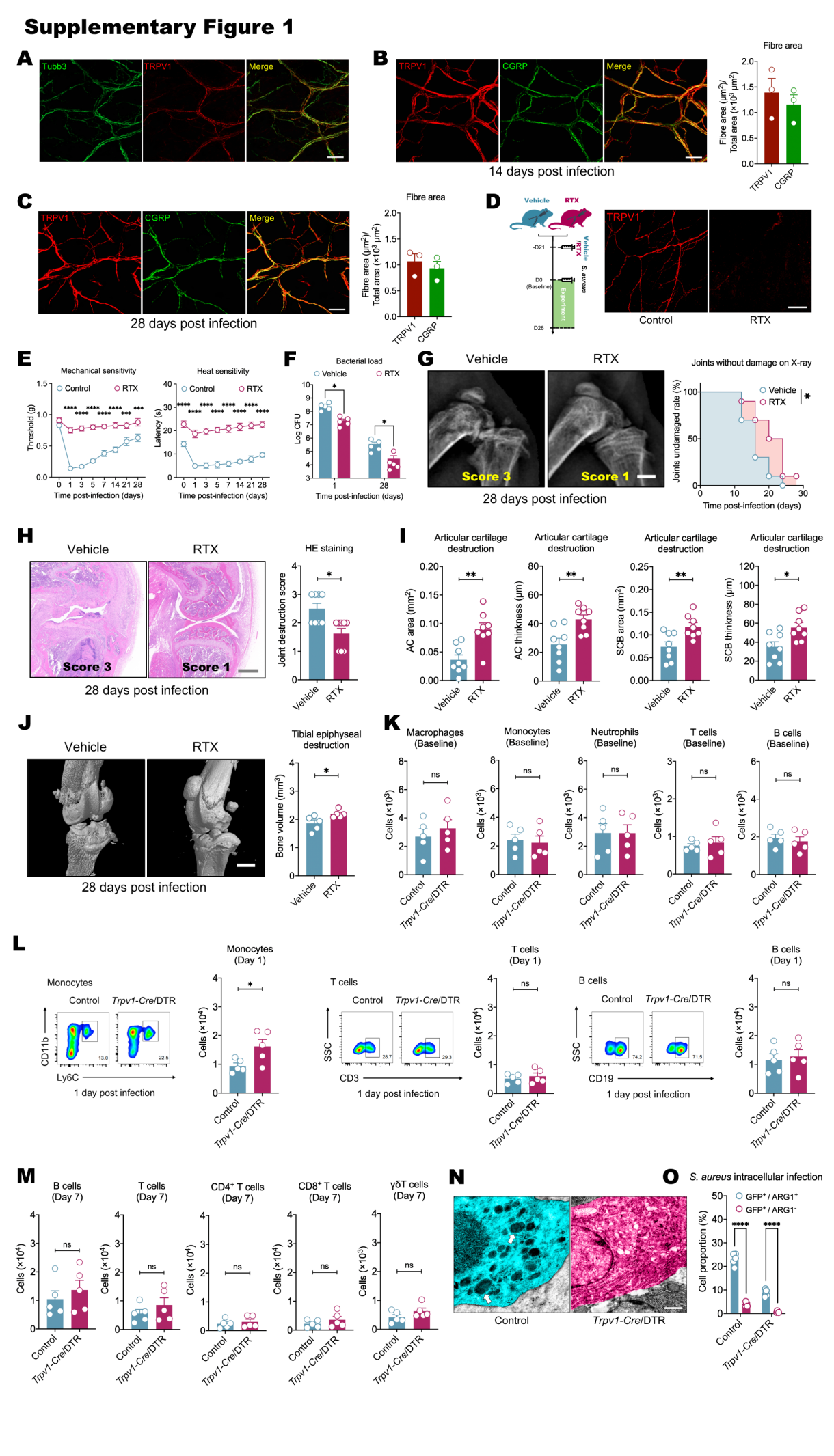


**Figure S1:**

1. Typical whole-mount immunofluorescence staining revealed the presence of TUBB3^+^ and TRPV1^+^ nerves in synovial tissue. (Scale bar: 50 μm.)
2. Typical whole-mount immunofluorescence staining revealed the presence of TRPV1^+^ and CGRP^+^ nociceptive nerves in synovial tissue on the 14th day postinfection. Quantification of TRPV1^+^ and CGRP^+^ stained areas in the synovium. (Scale bar: 50 μm.)
3. Typical whole-mount immunofluorescence staining revealed the presence of TRPV1^+^ and CGRP^+^ nociceptive nerves in synovial tissue on the 28th day postinfection. Quantification of TRPV1^+^ and CGRP^+^ stained areas in the synovium. (Scale bar: 50 μm.)
4. Typical nociceptive nerve staining in the synovium of vehicle- and RTX (100 ng/5 μl)-treated mice. (Scale bar: 100 μm.)
5. Differences in mechanical (left) and thermal (right) hyperalgesia between vehicle- and RTX-treated mice after *S. aureus* (1×10^8^ CFU) infection. The time point labeled '0' signifies the baseline condition prior to the onset of infection. n=10 mice/group.
6. Differences in local bacterial load on the 1st day (left) and 28th day (right) between vehicle- and RTX-treated mice after *S. aureus* infection. n=5 mice/group.
7. Typical X-ray images of knee joints on the 28th day after *S. aureus* infection in vehicle- and RTX-treated mice (left) and survival curves reflecting the differences in joint destruction progression after *S. aureus* infection between the two groups (right). The time point labeled '0' signifies the baseline condition prior to the onset of infection. n=10 mice/group. (Scale bar: 500 μm.)
8. Typical H&E-stained images of knee joints (left) and scores based on H&E staining showing differences in synovial and bone damage between the vehicle- and RTX-treated mice on the 28th day after *S. aureus* infection (right). n=8 mice/group. (Scale bar: 500 μm.)
9. Differences in the area and thickness of the articular cartilage (AC area and AC thickness) and subchondral bone (SCB area and SCB thickness) of the tibial plateaus between the two groups on the 28th day after S. aureus infection. n=8 mice/group.
10. Typical micro-CT images of knee joints on the 28th day after *S. aureus* infection in vehicle- and RTX-treated mice (left) and the differences in tibial epiphyseal volume between the two groups (right). n=5 mice/group. (Scale bar: 500 μm.)
11. Flow cytometry quantification of various immune cell populations in the knee joint synovium of control and *Trpv1-Cre/*DTR mice before infection. n=5 mice/group.
12. Flow cytometry quantification of various immune cell populations in the knee joint synovium of control and *Trpv1-Cre/*DTR mice on the 1st day after *S. aureus* infection. n=5 mice/group.
13. Flow cytometry quantification of various immune cell populations in the knee joint synovium of control and *Trpv1-Cre/*DTR mice on the 7th day after *S. aureus* infection. n=5 mice/group.
14. Typical transmission electron microscope image of macrophages and intracellular *S. aureus* in the knee joint synovium of control and *Trpv1-Cre/*DTR mice on the 28th day after infection. (Arrows indicate *S. aureus*; scale bar: 1 μm.)
15. Proportion of GFP^+^ macrophages in ARG1^+^ macrophages and ARG1^-^ macrophages on the 28th day after GFP^+^ *S. aureus* (1×10^8^ CFU) infection. n=5 mice/group.

Statistical analysis: (E, F, O) Two-way ANOVA, Sidak post hoc test. (I-M) Student’s t tests. (G) Restricted mean survival time (RMST). (H) Mann-Whitney U test. *p < 0.05, **p < 0.01, ***p < 0.001, ****p < 0.0001. ns = not significant. Mean ± SEM.


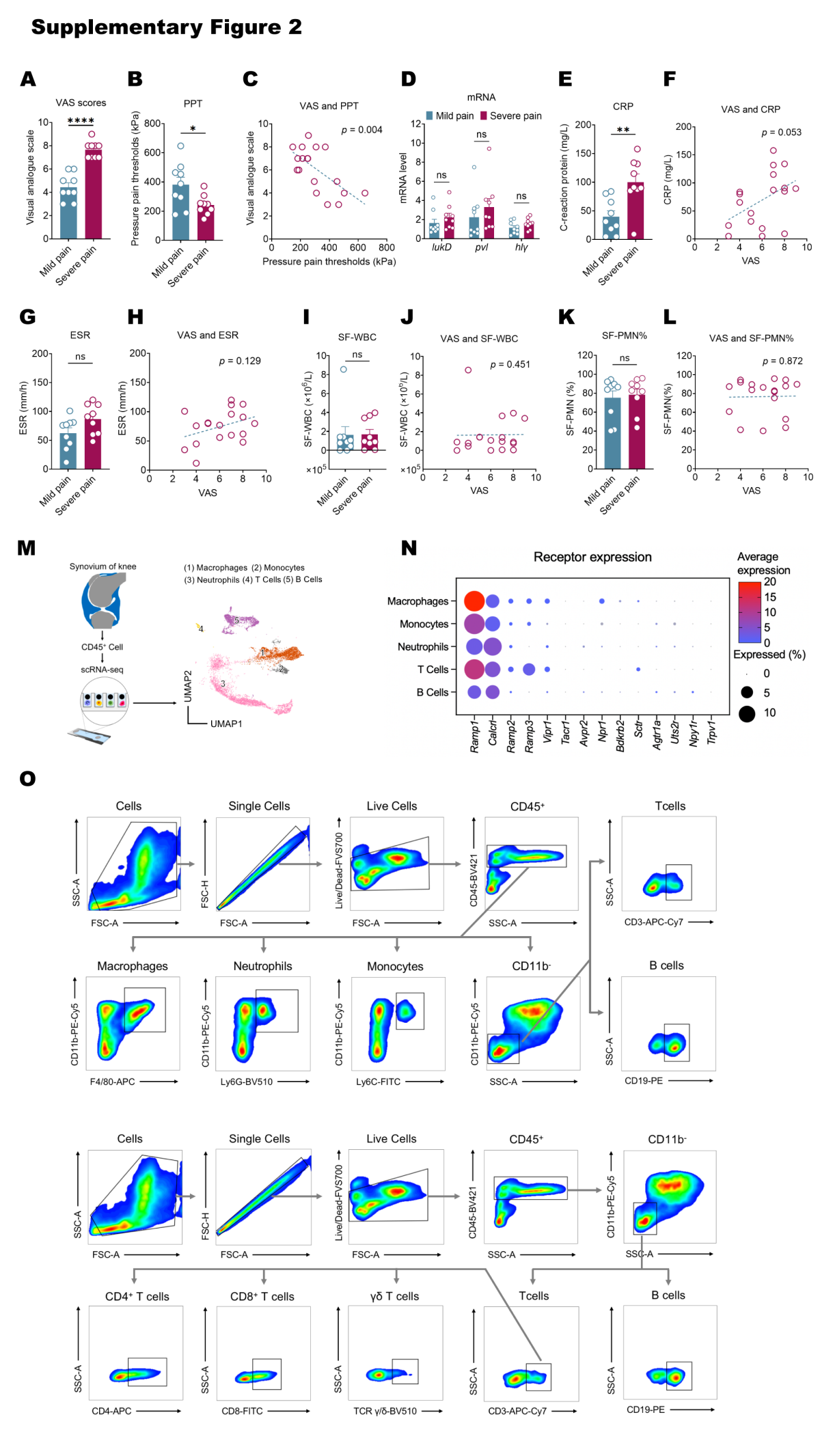


**Figure S2:**

1. Comparison of VAS scores between patients with severe pain (VAS score>6) and patients with mild pain (VAS score≤6). n=9 patients/group.
2. Comparison of corrected PPTs between the two groups of patients. n=9 patients/group.
3. Evaluation of the correlation between patient VAS scores and corrected PPTs. n=18, p=0.004.
4. qRT-PCR analysis comparing the expression of *the lukD*, *pvl*, and *hlγ* genes in *S. aureus* strains isolated from the two groups of patients. n=9 strains/group.
5. L) Comparison of differences in CRP (E), ESR (G), SF-WBC (I), and SF-PMN% (K) between the two groups of patients (n=9 patients/group) and analysis of the correlation between the four inflammatory indicators and VAS scores (n=18).

(M) Single-cell sequencing schematic of CD45^+^ cells from the uninfected synovium (left, saline injection for 24 h) and UMAP visualization to identify various cell types (right).

(N) Dot plots showing the average expression levels per immune cell cluster and the percentage of cells from each cluster expressing genes encoding neuropeptide receptors. n=10 pooled synovial samples.

(O) Gating strategy for flow cytometry.

Statistical analysis: (A, B, E, G, I, K) Student’s t tests. (C, F, H, J, L) Spearman rank test. (D) Two-way ANOVA, Sidak post hoc test. *p < 0.05, **p < 0.01, ***p < 0.001, ****p < 0.0001. ns = not significant. Mean ± SEM.


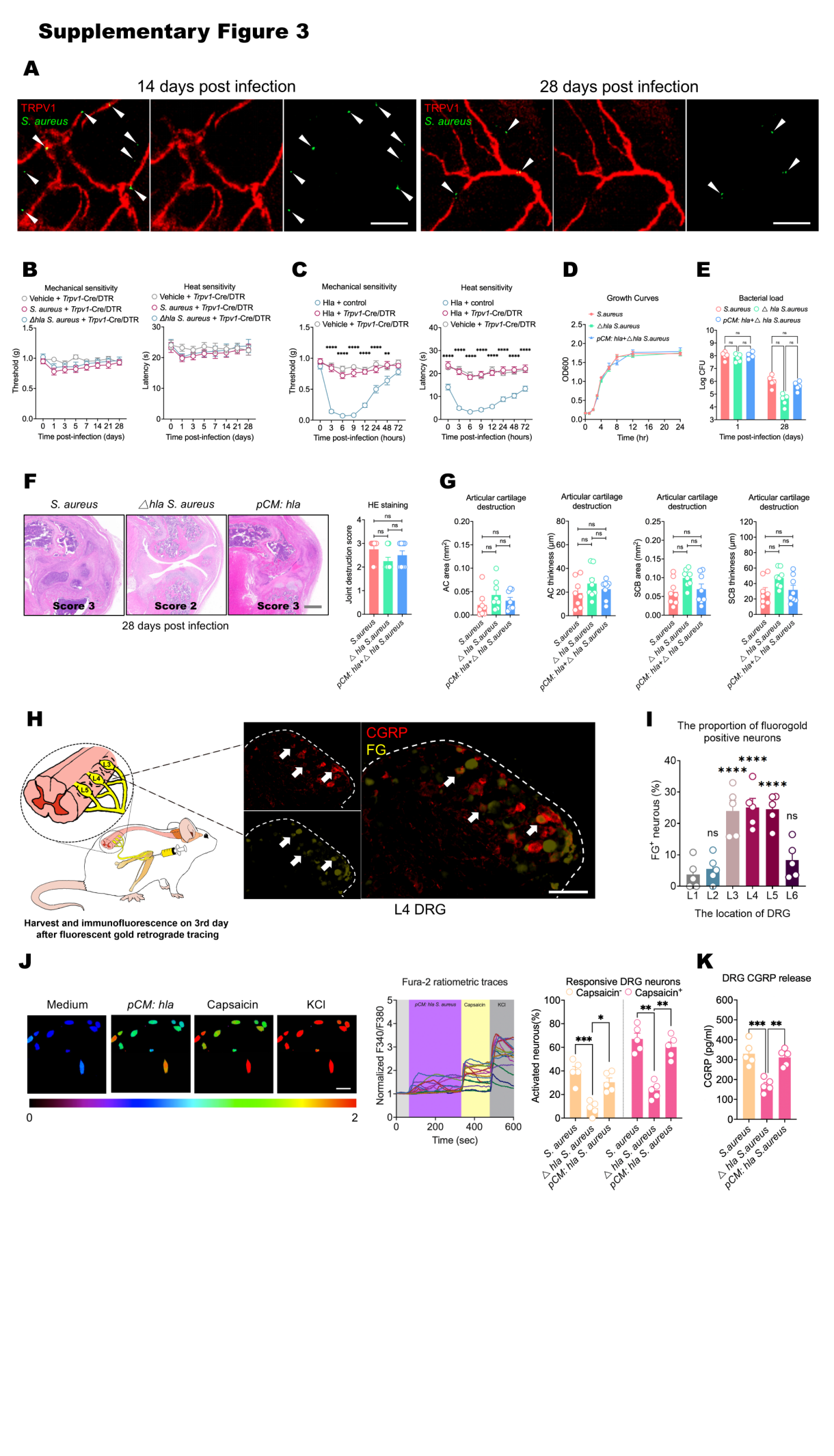


**Figure S3:**

(A) Whole-mount staining revealed contact between TRPV1^+^ nociceptive neurons (red) and *S. aureus* (green) at 14 days (left) and 28 days (right) after infection. (arrowheads: GFP^+^ *S. aureus*). (Scale bar: 10 μm.)

(B) Differences in mechanical (left) and thermal (right) hyperalgesia among vehicle-, *S. aureus* (1×10^8^ CFU)-, and *Δhla-S. aureus* (1×10^8^ CFU)-infected *Trpv1*-Cre/DTR mice. The time point labeled '0' signifies the baseline condition prior to the onset of infection. n=10 mice/group.

(C) Differences in mechanical (left) and thermal (right) hyperalgesia among Hla-treated control mice, Hla-treated *Trpv1*-Cre/DTR mice, and vehicle-treated *Trpv1*-Cre/DTR mice. The time point labeled '0' signifies the baseline condition prior to the onset of infection. n=10 mice/group.

(D) Growth curves of different *S. aureus* strains. n=5 samples/group.

1. Bacterial load in samples collected on the 1st day and 28th day postinjection of *S. aureus* from *S. aureus*-infected mice, *△hla*-*S. aureus*-infected mice and *pCM: hla*+*△hla*-*S. aureus*-infected mice. n=5 mice/group.
2. Typical H&E-stained images of knee joints (left) and scores based on H&E staining showing differences in synovial and bone damage among the three groups on the 28th day after *S. aureus* infection (right). n=8 mice/group. (Scale bar: 500 μm).
3. Differences in the area and thickness of the articular cartilage (AC area and AC thickness) and subchondral bone (SCB area and SCB thickness) of the tibial plateaus among the three groups on the 28th day after S. aureus infection. n=8 mice/group.
4. Schematic diagram of mouse knee joint FG retrograde tracing and DRG collection (left). Immunofluorescence staining of L4 DRGs demonstrated the colocalization of FG with CGRP in neurons (right). (arrows: FG^+^ CGRP^+^ cells; scale bar: 100 μm).
5. CGRP^+^ neurons innervating the knee joint are located primarily in the L3-L5 DRGs.
6. Calcium imaging of DRG neurons stimulated with *pCM: hla*+*Δhla*-*S. aureus* (1×10^9^ CFU) and capsaicin (1 µM), showing typical fields of view (left) and calcium traces (middle). (Scale bar: 50 μm; color bar, F340/F380 ratio). The right graph represents the proportions of capsaicin-nonresponsive and capsaicin-responsive neurons activated by *S. aureus* (1×10^9^ CFU), *Δhla-S. aureus* (1×10^9^ CFU) and *pCM: hla*+*Δhla*-*S. aureus* (1×10^9^ CFU). n=5 wells/group.
7. ELISA of CGRP release in the cell culture supernatants of DRG neurons stimulated with *S. aureus* (1×10^9^ CFU), *Δhla-S. aureus* (1×10^9^ CFU) or *pCM: hla*+*Δhla*-*S. aureus* (1×10^9^ CFU) for 30 min. n=5 wells/group.

Statistical analysis: (B-E) Two-way ANOVA with Sidak post hoc test. (F) Kruskal-Wallis H test with Dunn's post hoc test. (G, I, K) One-way ANOVA, Dunnett post hoc test. *p < 0.05, **p < 0.01, ***p < 0.001, ****p < 0.0001. ns = not significant. Mean ± SEM.


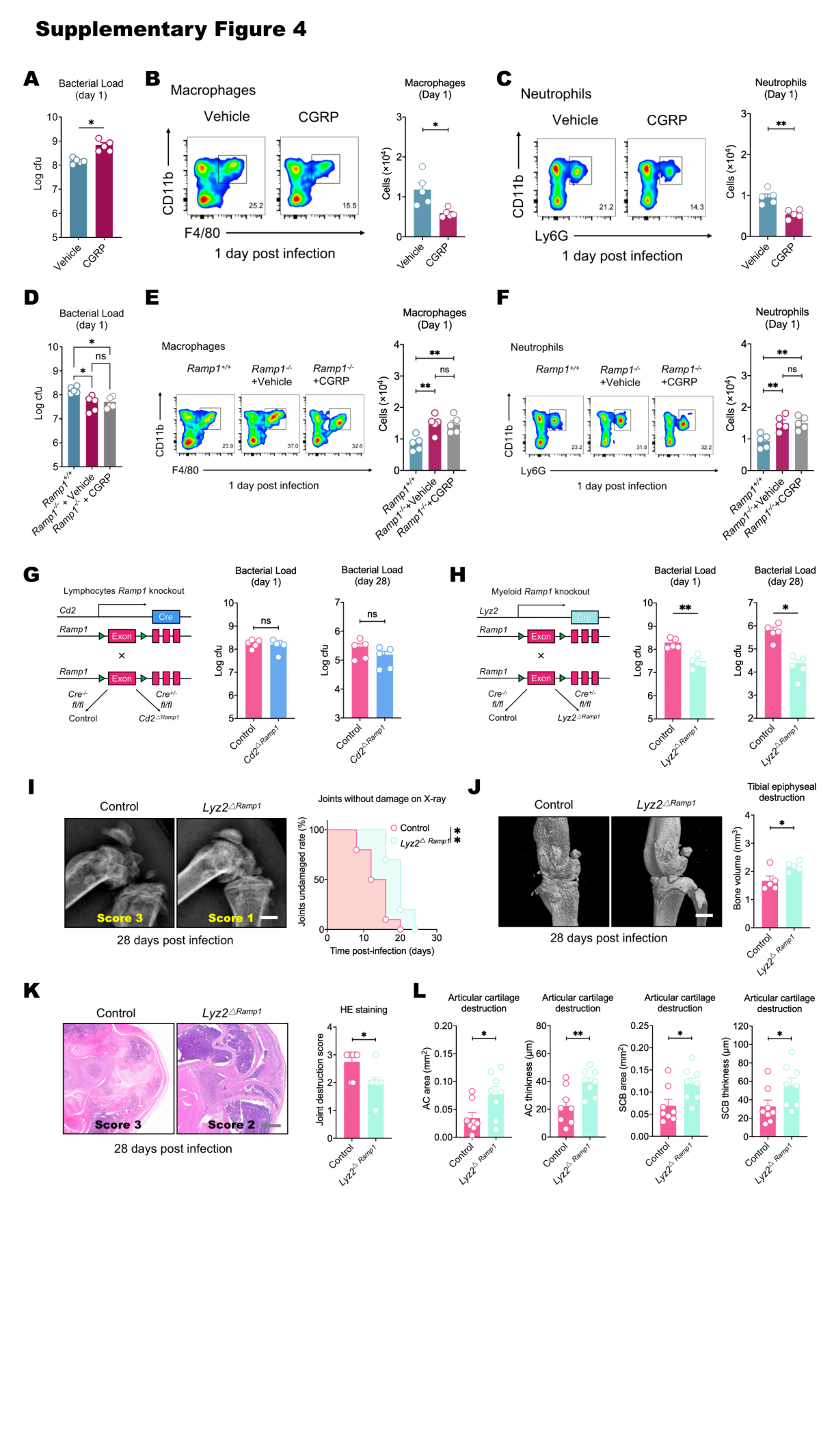


**Figure S4:**

1. Local bacterial load differences between vehicle-treated mice and CGRP (2 μg/5 μl)-treated mice on the 1st day post infection. n=5 mice/group.
2. Representative flow cytometry images and quantification of macrophages in the knee synovium of vehicle-treated mice and CGRP-treated mice on the 1st day post infection. n=5 mice/group.
3. Representative flow cytometry images and quantification of neutrophils in the knee synovium of vehicle-treated mice and CGRP-treated mice on the 1st day post infection. n=5 mice/group.
4. Local bacterial load differences among the control, RAMP1 receptor knockout (*Ramp1^-/-^*) combined with vehicle-treated and Ramp1 receptor knockout combined with CGRP-treated groups on the 1st day post infection. n=5 mice/group.
5. Representative flow cytometry images and quantification of macrophages in the knee synovium of *Ramp1^+/+^*, vehicle-treated *Ramp1^-/-^* and CGRP-treated *Ramp1^-/-^* mice on the 1st day post infection. n=5 mice/group.
6. Representative flow cytometry images and quantification of neutrophils in the knee synovium of *Ramp1^+/+^*, vehicle-treated *Ramp1^-/-^* and CGRP-treated *Ramp1^-/-^* mice on the 1st day post infection. n=5 mice/group.
7. Generation of lymphocytes-specific *Ramp1*-knockout (*Cd2^ΔRamp1^*) mice (left). Differences in the local bacterial load of the knee joint on the 1st day (middle) and 28th day (right) post infection between control and *Cd2^ΔRamp1^* mice. n=5 mice/group.
8. Generation of myeloid immune cell-specific *Ramp1*-knockout (*Lyz2^ΔRamp1^*) mice (left). Differences in the local bacterial load of the knee joint on the 1st day (middle) and 28th day (right) post infection between control and *Lyz2^ΔRamp1^* mice. n=5 mice/group.
9. Typical X-ray images of knee joints on the 28th day after *S. aureus* infection in control and *Lyz2^ΔRamp1^* mice (left) and survival curves reflecting the differences in bone destruction progression between the two groups (right). n=10 mice/group. (Scale bar: 500 μm.)
10. Typical micro-CT images of knee joints on the 28th day after *S. aureus* infection in control and *Lyz2^ΔRamp1^* mice (left) and the differences in tibial epiphyseal volume between the two groups (right). n=5 mice/group. (Scale bar: 500 μm.)
11. Typical H&E-stained images of knee joints (left) and scores based on H&E staining showing differences in synovial and bone damage between the two groups on the 28th day after *S. aureus* infection (right). n=8 mice/group. (Scale bar: 500 μm.)
12. Differences in the area and thickness of the articular cartilage (AC area and AC thickness) and subchondral bone (SCB area and SCB thickness) of the tibial plateaus between the two groups on the 28th day after S. aureus infection. n=8 mice/group.

Statistical analysis: (A, B, C, G, H, J, L) Student’s t tests. (D, E, F) One-way ANOVA with Tukey’s post hoc test. (I) Restricted mean survival time test. (K) Mann-Whitney U test. *p < 0.05, **p < 0.01, ***p < 0.001, ****p < 0.0001. ns = not significant. Mean ± SEM.


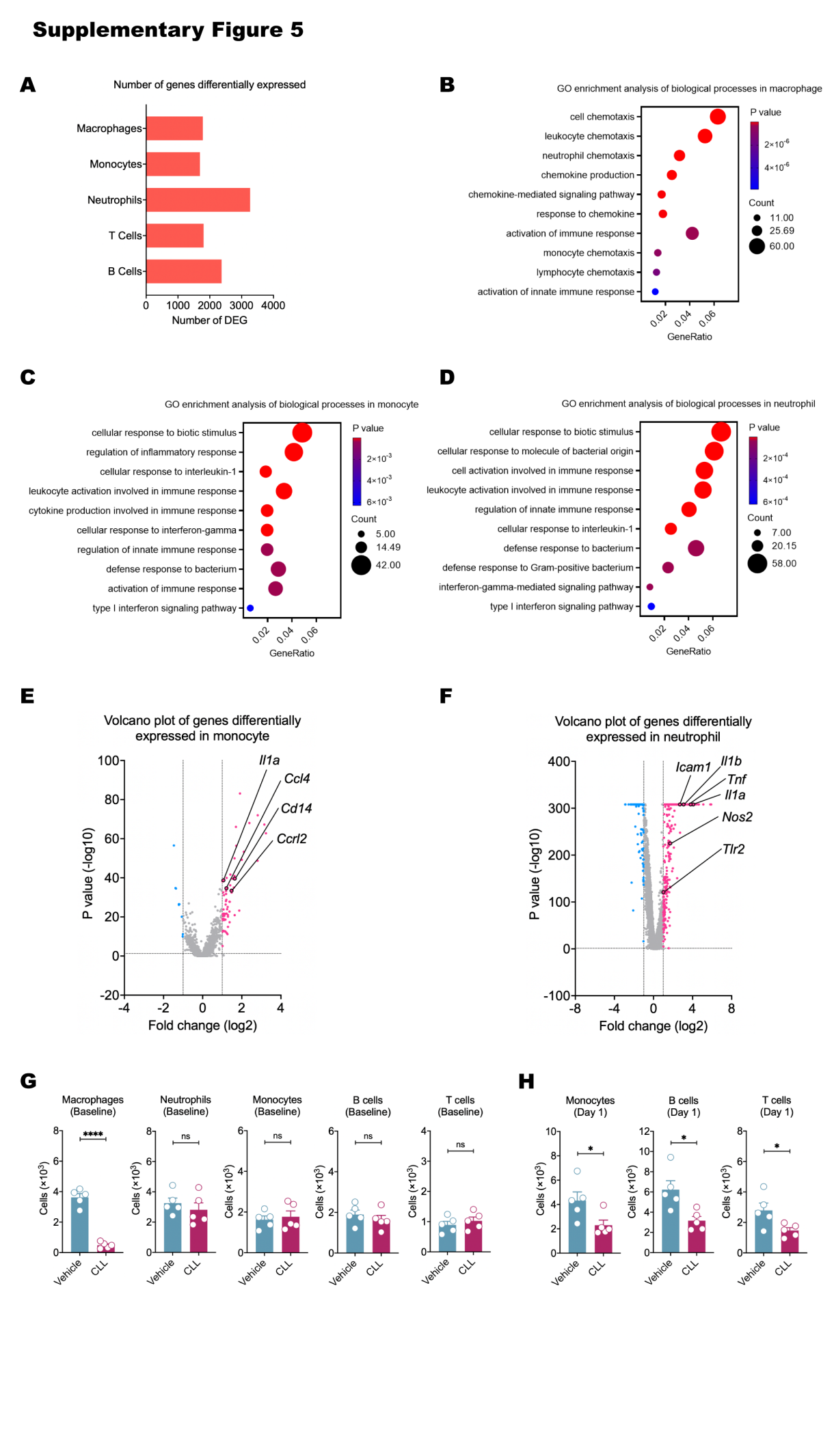


**Figure S5:**

(A) Numbers of differentially expressed genes in various immune cell populations isolated from infected mouse synovium compared with those in the corresponding populations from uninfected mouse synovium. n=10 pooled synovium/group.

(B-D) Bubble charts showing GO biological process enrichment analysis of upregulated differentially expressed genes in macrophages (B), monocytes (C), and neutrophils (D), highlighting chemotaxis- and anti-infection-related biological processes. n=10 pooled synovium/group.

(E-F) Volcano plots displaying differentially expressed genes in monocytes (E) and neutrophils (F) between infected and uninfected mouse synovium, emphasizing anti-infection-related upregulated genes. The red dots represent upregulated DEGs, the blue dots represent downregulated DEGs, and the gray dots represent genes whose expression was not significantly different. n=10 pooled synovium/group.

(G) Flow cytometric quantification of synovial macrophages, monocytes, neutrophils, and B and T cells 3 days after intra-articular injection of CLLs (5 µl) or vehicle. n=5

(H) Flow cytometric quantification of synovial monocytes and B and T cells 1 day after *S. aureus* (1×10^8^ CFU) infection in mice treated with CLLs or vehicle.

Statistical analysis: (G, H) Student’s t tests. *p < 0.05, ****p < 0.0001. ns = not significant. Mean ± SEM.


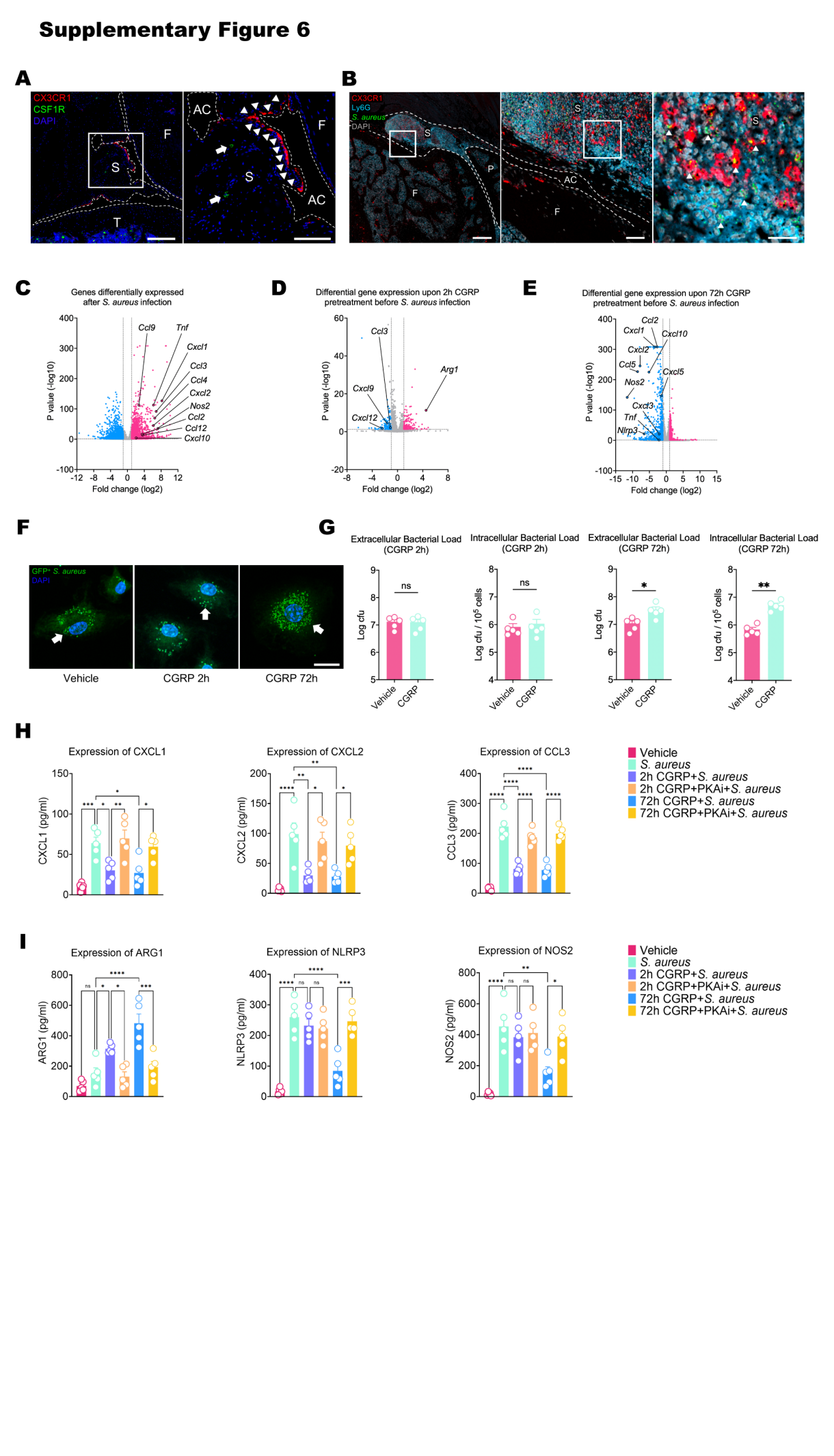


**Figure S6:**

(A) Sagittal section immunofluorescence staining of knee joints showing the distribution of CX3CR1^+^ lining macrophages and CSF1R^+^ interstitial macrophages in the synovium (arrows: CSF1R^+^ interstitial macrophages; arrowheads: CX3CR1^+^ lining macrophages; T: tibia; F: femur; AC: articular cavity; S: synovium; scale bar: 200 μm, 100 μm).

(B) Representative images at 48 h postinfection showing a scattered distribution of GFP^+^ *S. aureus* throughout the synovial tissue, whereas CX3CR1^+^ synovial macrophages and recruited Ly6G^+^ neutrophils were diffusely distributed in the synovium, unlike in the initial stage of infection (arrowheads: *S. aureus*; F: femur; AC: articular cavity; S: synovium; scale bar: 300 μm, 50 μm, 20 μm).

(C‒E) Volcano plots displaying differentially expressed genes in synovial macrophages between the infected group and uninfected group (C), between the infected group and the 2 h CGRP (100 nM)-pretreated infected group (D), and between the infected group and the 72 h CGRP-pretreated infected group (E) (*S. aureus* MOI=100). The red dots represent upregulated DEGs, the blue dots represent downregulated DEGs, and the gray dots represent genes whose expression was not significantly different. n=5 mice/group.

(F) Representative images showing the survival of intracellular *S. aureus* (arrows, GFP^+^) within synovial macrophages in the 2 h and 72 h CGRP-pretreated groups. (Scale bar: 20 μm.)

(G) Differences in extracellular and intracellular bacterial loads at 24 h post *S. aureus* infection (MOI=100) between control and CGRP-pretreated (2 h and 72 h) synovial macrophages. n=5 wells/group.

(H) Concentrations of chemotactic cytokines in macrophages after 24 h of incubation with vehicle, *S. aureus* (MOI=100), CGRP (100 nM) pretreated for 2 h + *S. aureus*, PKAi (10 μM) + CGRP pretreated for 2 h+ *S. aureus*, CGRP pretreated for 72 h + *S. aureus*, or PKAi + CGRP pretreated for 72 h + *S. aureus*. n=5 wells/group.

(I) Concentrations of ARG1, NLRP3 and NOS2 in macrophages after 24 h of incubation with vehicle, *S. aureus* (MOI=100), CGRP (100 nM) pretreated for 2 h + *S. aureus*, PKAi + CGRP pretreated for 2 h+ *S. aureus*, CGRP pretreated for 72 h + *S. aureus*, or PKAi + CGRP pretreated for 72 h + *S. aureus*. n=5 wells/group.

Statistical analysis: (G) Student’s t tests. (H, I) One-way ANOVA with Tukey’s post hoc test. *p < 0.05, **p < 0.01, ***p < 0.001, ****p < 0.0001. ns = not significant. Mean ± SEM.


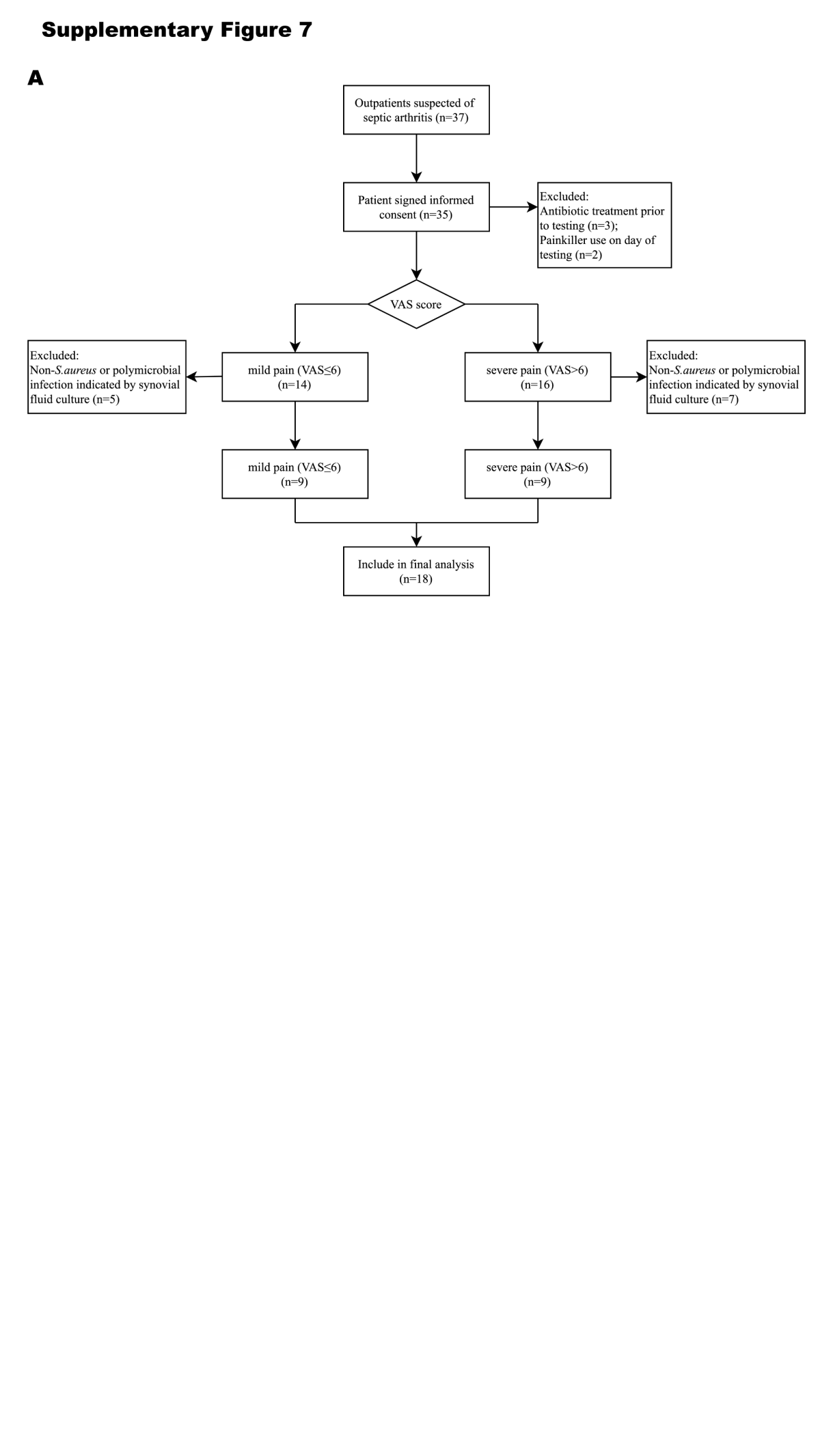


**Figure S7:**

(A) Patient selection process for the study. The flowchart depicts the process of inclusion and exclusion of patients suspected of having septic arthritis. A total of 37 patients were initially suspected, 18 of whom were ultimately included in the study. The reasons for exclusion (such as non-*S. aureus* infection, prior antibiotic use, or painkiller use on test day) are detailed.

**Table S1**

Comparative Analysis of Demographic Characteristics Across the Two Groups

|  | Mild Pain Group | Severe Pain Group | *P* Value |
| --- | --- | --- | --- |
| Gender: Male (%) | 8 (88.9%) | 7 (77.8%) | 1.000^$^ |
| Age (Years, mean ± SD) | 56.6 ± 14.7 | 51.1 ± 16.6 | 0.473^&^ |
| BMI (kg m^-2^, mean ± SD) | 23.1± 4.1 | 24.1± 2.0 | 0.622^&^ |
| aCCI (mean (IQR)) | 2 (3) | 2 (3) | 0.587^#^ |
| Affected Side: Left (%) | 5 (55.6%) | 5 (55.6%) | 1.000^$^ |
| Fever: Present (%) | 3 (33.3%) | 3 (33.3%) | 1.000^$^ |
| MRSA (%) | 2 (22.2%) | 4 (44.4%) | 0.620^$^ |

BMI: body mass index; aCCI: age-adjusted Charlson comorbidity index; MRSA: methicillin-resistant *Staphylococcus aureus*. Continuous variables with a normal distribution are presented as the means ± SDs (standard deviations); nonnormally distributed variables are expressed as medians (IQRs - interquartile ranges); and categorical variables are denoted as totals (percentages). The symbol '&' indicates results from the independent samples t test; '#' indicates results from the Mann‒Whitney U test; and '$' indicates results from Fisher's exact test.
